# Amygdala Modulation During Emotion Regulation Training With fMRI-Based Neurofeedback

**DOI:** 10.3389/fnhum.2019.00089

**Published:** 2019-03-26

**Authors:** Ana Rita Barreiros, Inês Almeida, Bárbara Correia Baía, Miguel Castelo-Branco

**Affiliations:** CIBIT, ICNAS–Institute of Nuclear Sciences Applied to Health-and CNC.IBILI–Faculty of Medicine, Coimbra Institute for Biomedical Imaging and Translational Research, University of Coimbra, Azinhaga de Santa Comba, Coimbra, Portugal

**Keywords:** amygdala, emotion regulation, fMRI, neurofeedback, systematic review

## Abstract

Available evidence suggests that individuals can enhance their ability to modulate brain activity in target regions, within the Emotion Regulation network, using fMRI-based neurofeedback. However, there is no systematic review that investigates the effectiveness of this method on amygdala modulation, a core region within this network. The major goal of this study was to systematically review and analyze the effects of real-time fMRI-Neurofeedback concerning the neuromodulation of the amygdala during Emotion Regulation training. A search was performed in PubMed, Science Direct, and Web of Science with the following key terms: ≪(“neurofeedback” or “neuro feedback” or “neuro-feedback”) and (“emotion regulation”) and (fMRI OR “functional magnetic resonance”),≫ and afterwards two additional searches were performed, replacing the term “emotion regulation” for “amygdala” and “neurofeedback” for “feedback.” Of the 531 identified articles, only 19 articles reported results of amygdala modulation during Emotional Regulation training through rtfMRI-NF, using healthy participants or patients, in original research articles. The results, systematically reviewed here, provide evidence for amygdala's modulation during rtfMRI-NF training, although studies' heterogeneity precluded a quantitative meta-analysis—the included studies relied on different outcome measures to infer the success of neurofeedback intervention. Thus, a qualitative analysis was done instead. We identified critical features influencing inference on the quality of the intervention as: the inclusion of a Practice Run, a Transfer Run and a Control Group in the protocol, and to choose adequate Emotion Regulation strategies—in particular, the effective recall of autobiographic memories. Surprisingly, the Regulated vs. Control Condition was lacking in most of the studies, precluding valid inference of amygdala neuromodulation within Session. The best controlled studies nevertheless showed positive effects. The type of stimulus/interface did not seem critical for amygdala modulation. We also identified potential effects of lateralization of amygdala responses following Up- or Down-Regulation, and the impact of fMRI parameters for data acquisition and analysis. Despite qualitative evidence for amygdala modulation during rtfMRI-NF, there are still important limitations in the design of a clear conceptual framework of NF-training research. Future studies should focus on more homogeneous guidelines concerning design, protocol structure and, particularly, harmonized outcome measures to provide quantitative estimates of neuromodulatory effects in the amygdala.

## Introduction

Emotion Regulation (ER) plays a vital role in adaptive behavior. The ability to regulate emotional responses is essential in ensuring productivity in working environments and in social adaptation. Dysfunction in this ability is common in many of psychiatric diseases, including social anxiety disorder (Jazaieri et al., 2015), major depressive disorder (Yuan et al., 2014; Stephanou et al., 2017), eating disorders (Donofry et al., 2016), personality disorders (van Zutphen et al., 2015), or in autism spectrum disorders (Weiss et al., 2014).

A better understanding of the neural underpinnings of ER processes is of great interest for the development of new therapeutic interventions. According to a cognitive control model of ER, its neural implementation can be summarized as resulting from the interactions of the prefrontal (PFC) and the anterior cingulate (ACC) cortical regions and their influence on subcortical structures, in particular the amygdala (Sergerie et al., 2008; Zilverstand et al., 2016; Stephanou et al., 2017). Despite the complex interactions within the ER network, it is widely accepted that the amygdala is a critical region for the generation, expression, and experience of emotions (LeDoux, 2007; Duvarci and Pare, 2014; Frank et al., 2014). Therefore, therapeutic strategies targeting this structure are of major importance.

Neurofeedback (NF) draws on multiple techniques that allows self-regulation enhancement of neural activity in health and disease. It implies the use of non-invasive training methods for the self-regulation of neural activity to alter neural plasticity and learning, by providing individuals with real time information about their brain responses (Sulzer et al., 2013; Stoeckel et al., 2014). These techniques continue to be a focal point of development of possible treatments for neuropsychiatric disorders. Specifically, real-time functional magnetic resonance imaging neurofeedback (rtfMRI-NF) has been revealed as a promising and useful clinical tool to enhance individual ER capability in neuropsychiatric disorders (Johnston et al., 2010; Zotev et al., 2011; Young et al., 2014; Brühl, 2015;Emmert et al., 2016).

Importantly, when assessing new therapeutic approaches, it is necessary to consider both general and disorder-specific deficits, in order to select the appropriate neural targets. For instance, when planning interventions targeting particular deficits or psychiatric symptoms related with dysregulation of emotional processes, fMRI-based NF can first use functional localizer scans that allow for a specific choice of target areas within the core regulatory network of ER (Johnston et al., 2010). However, given that the amygdala is easy to reliably identify from the anatomical point of view, a functional localizer is not mandatory in this case.

To date, several studies have demonstrated that individuals can enhance their ability to modulate their own brain activity, by using rtfMRI-NF approaches that target networks that are relevant to voluntary ER (Paret et al., 2016; Nicholson et al., 2017). Most studies that address the modulation of the emotional network, define the amygdala as the main target (Costafreda et al., 2008; Zotev et al., 2011; Brühl et al., 2014; Paret et al., 2014, 2016; Keynan et al., 2016) based on evidence that ER involves the modulation of amygdala activity (Ochsnet et al., 2012). Accordingly, several studies that did not use rtfMRI, have addressed the importance of the amygdala during ER and cognitive reappraisal of emotion (e.g., Sarkheil et al., 2015; for a meta-analysis see Buhle et al., 2013) and mood induction (e.g., Dyck et al., 2011; Kohn et al., 2014). The success of interventions focusing the control of brain areas during ER training, including the amygdala, would provide confirmatory information that one's emotional state, at least to some extent, is capable of being modulated, and this might have an important clinical impact.

However, there has been some controversy around the results reported thus far, due to heterogeneity among studies in terms of outcome measures, characteristics of the applied protocol, or the included participants (Sulzer et al., 2013; Thibault et al., 2018), further extended by the debate on what constitutes a proper control condition (Sorger et al., 2019). Commonly, NF training protocols include a recorded Baseline of brain activity, sometimes followed by a Localizer Run (to functionally define the target Region-of-interest (ROI) unless an anatomically defined ROI is chosen (Sulzer et al., 2013), or when data-driven approaches are not used instead (e.g., LaConte et al., 2007; LaConte, 2011) and Training Runs. During the Training Runs, the participant practices the ER task with feedback of the brain signal in real time. Particularly, the participant should apply ER strategies to increase (Up-Regulate) or decrease (Down-Regulate) the activation of the targeted brain region (Stoeckel et al., 2014). In some studies, a Practice Run precedes the NF Training Runs. Moreover, studies may also include a Transfer Run at the end of one or multiple Sessions (Sulzer et al., 2013). In this Transfer Run, the participant is instructed to regulate brain activation as in prior Training Runs, but without receiving any feedback (Zotev et al., 2011; Sulzer et al., 2013; Stoeckel et al., 2014). In these cases, the Transfer Run is included in the protocols to understand if the participant can learn to self-regulate the target brain region's activity without NF (Zotev et al., 2011; Stoeckel et al., 2014) and to translate it for the outside world (Sulzer et al., 2013). Most studies employ only a single Session, but NF training can be performed throughout several Sessions (Sulzer et al., 2013), which allows for mental training between Sessions (Subramanian et al., 2011; Sulzer et al., 2013) and positive carry-over effects (Rieger et al., 2018).

Besides the heterogeneity of NF studies in terms of protocols and outcome measures, it is important to identify other factors that may affect such outcomes when using the amygdala as the target region. For instance, the known rapid habituation of this structure even to emotionally loaded faces (Breiter et al., 1996) may represent a limitation in NF approaches because it limits the potential for neuromodulation. In addition, echoplanar imaging (EPI) of this region is known to create susceptibility-induced magnetic field inhomogeneities with consequent signal loss and geometric distortion (Merboldt et al., 2001; Olman et al., 2009). Several studies have tested optimizations for fMRI sequences targeting the amygdala, and reported optimal values in terms of echo time (TE), voxel size, slice thickness and section orientation (Merboldt et al., 2001; Chen et al., 2003; Posse et al., 2003a,b; Robinson et al., 2004, 2005; Morawetz et al., 2008; Olman et al., 2009). All these approaches have tradeoffs that need to be carefully considered when estimating final signal to noise ratios, and statistical effect sizes. Some authors advise the use of multi-echo instead of single-echo acquisitions when measuring amygdala activation (Posse et al., 2003a,b), as it increases sensitivity to the BOLD response through a better contrast-to-noise ratio (Caballero-Gaudes and Reynolds, 2017). However, this may imply limited brain volume coverage (Posse et al., 2003b). But method studies using single-echo EPI acquisitions have also reported good results (e.g., (Kirilina et al., 2016)), while others have suggested the reduction of section thickness to improve the detectability of amygdala activation (Bellgowan et al., 2006; Morawetz et al., 2008). The use of shorter repetition times (TRs) to minimize physiological signal confounds such as blood pulsation in Blood-Oxygen-Level Dependent (BOLD) signal changes (Posse et al., 2003a,b; Caballero-Gaudes and Reynolds, 2017) has also been suggested but again at the cost of full brain coverage (Caballero-Gaudes and Reynolds, 2017), as well as possibly introducing other unwanted noise (Zhao et al., 2000).

Importantly, data denoising methods should be considered in advance, with concomitant measurements of physiological signals such as heart and respiratory rate during the rtfMRI-NF data acquisition (Caballero-Gaudes and Reynolds, 2017). By improving signal-to-noise ratio (SNR), through the reduction of both instrumental and physiological noise, relative effect sizes increase and consequently, sensitivity becomes improved. The use of techniques such as parallel imaging acceleration (e.g., SENSE, sensitivity encoding; and GRAPPA, generalized autocalibrating partially parallel Acquisition) (Blaimer et al., 2004; Bhavsar et al., 2013) to reduce the costs of EPI—geometric distortion and signal loss—in regions such as the amygdala (Olman et al., 2009; Bhavsar et al., 2013) is becoming more common, particularly in rtfMRI-NF. Although these techniques may impact SNR (Olman et al., 2009), technological improvements and the use of arrayed instead of birdcage coils (de Zwart et al., 2004; Bellgowan et al., 2006) have shown enhancement of temporal SNR (tSNR) and contrast-to-noise ratio (CNR) in medial temporal regions (Bellgowan et al., 2006).

In summary, given its cost and complexity, it is crucial to demonstrate how rtfMRI-NF training can produce novel ER related task strategies for practical applications. It is therefore important to systematically review and analyze the effectiveness of this technique in the modulation of specific target areas, as well as effects sizes (Rogala et al., 2016). Additionally, the study of specific brain activity patterns related to ER may help elucidate further about its neural underpinnings. Consequently, it motivates the optimization of rtfMRI-NF techniques for the training of ER abilities.

Here, we systematically reviewed the reported evidence of regulating amygdala activity with rtfMRI-NF during ER training. Given the potential relevance of NF in basic and clinical neuroscience of ER and its possible therapeutic application in a wide range of neuropsychiatric disorders, the major goal of this review is to systematically assess and analyze the effects of rtfMRI-NF in the neuromodulation of the amygdala during ER training. We aimed to collect evidence from available studies that reported changes in brain activity and to discuss the reported effectiveness of this technique.

The main objectives of this revision were: (1) the definition (characteristics) of NF protocols and (2) how the amygdala responds to ER training using rtfMRI-NF? Particularly, in the first point, we asked: (1.1) What were the outcome measures used among studies? (1.2) Were there sustained brain changes in the amygdala as a result of rtfMRI-NF neuromodulation across training runs (within the same Session)? (1.3) Were there sustained brain changes as a result of rtfMRI-NF neuromodulation training across different Sessions? (1.4) Were the brain changes only visible in the training group (compared to the control group)? and (1.5) Did the training effect depend on amygdala lateralization? In the second point, we defined questions as: (2.1) Which stimuli were used to induce emotional states and how effective were they? (2.2) Which ER strategies were the participants instructed to use and how effective were they? (2.3) How do fMRI acquisition, preprocessing and data analysis parameters relate to the training effects? (2.4) Was there any potential bias created by the NF protocols?

To answer these questions, we conducted a systematic review to analyze the potential quantitative and qualitative associations between the studies' characteristics and their reported results.

## Methods

### Systematic Review

#### Data Sources and Literature Search

**Figure 1:**
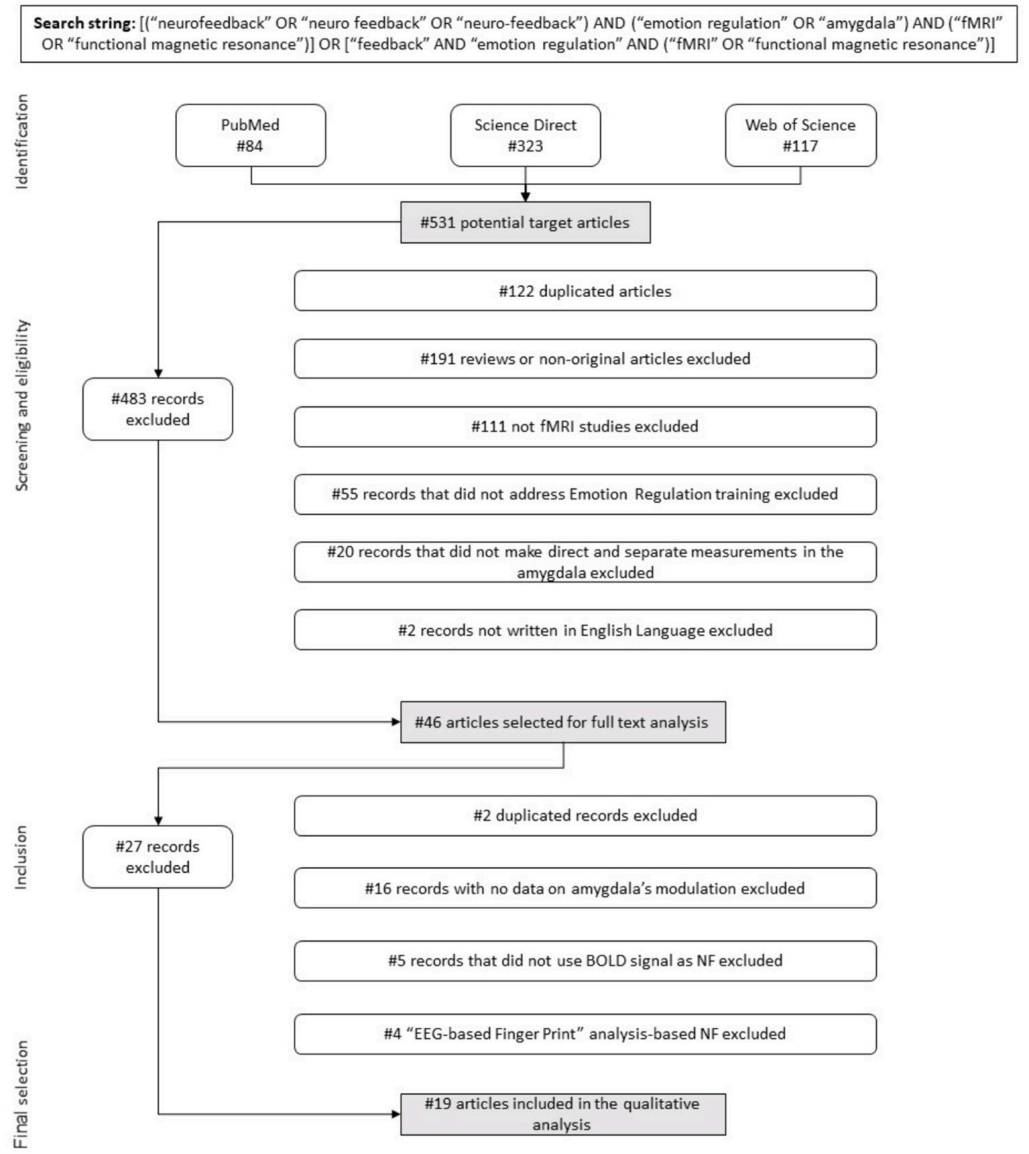
Flow diagram. Flow of information describing the different phases of the systematic review.

A systematic literature search was performed to identify studies that have investigated the training of ER through rtfMRI-NF, and to measure these training effects on amygdala blood-oxygen-level-dependent (BOLD) signals. This review was performed following the principles of the PRISMA statement (Liberati et al., 2009; Moher et al., 2009). This process underlies four phases: identification, screening, eligibility and inclusion (Figure 1). The research papers selected and reviewed in this study were identified through a search in the following databases: MEDLINE, via PubMed (http://www.ncbi.nlm.nih.gov/pubmed), Science Direct (Elsevier, http://www.sciencedirect.com/), and Web of Science (https://webofknowledge.com/). First, the following search string was used: (“neurofeedback” OR “neuro feedback” OR “neuro-feedback”), (“emotion regulation”), and (“fMRI” OR “functional magnetic resonance”). Afterwards, a second search was performed using the same combination of keywords but replacing “emotion regulation” with “amygdala,” in order to cover articles that presented data on amygdala responses to training in ER with NF, but using terms other than “emotion regulation.” Finally, a third search string was used, considering “emotion regulation,” (“fMRI” OR “functional magnetic resonance”), and using “feedback” instead of “neurofeedback.”

The search reported here was undertaken in September 2018, without imposing any publication date limit. Therefore, it includes articles published up to September 2018. In addition, references included in the articles deemed appropriate for full text revision were hand-searched to retrieve further relevant publications.

#### Eligibility Criteria, Screening Phase, and Study Selection

Studies adhering to the following criteria were considered eligible: (1) written in English; (2) involving adult human participants (animal studies were excluded); (3) involving original research articles (e.g., review articles were excluded); (4) applied rtfMRI-NF training (and not other forms of Neurofeedback, e.g., electroencephalography (EEG) neurofeedback or others), (5) directly addressed “emotion regulation” as the target cognitive process to modulate (and not other cognitive process), (6) made direct and separate measurements in the amygdala (either this is defined or not as the target region).

The study selection was performed in two different stages: a screening phase, in which titles and abstracts were analyzed and studies filtered based on the eligibility criteria, and a final selection phase, in which the full texts were analyzed. During the screening phase, duplicates were eliminated.

#### Data Extraction

**Figure 2:**
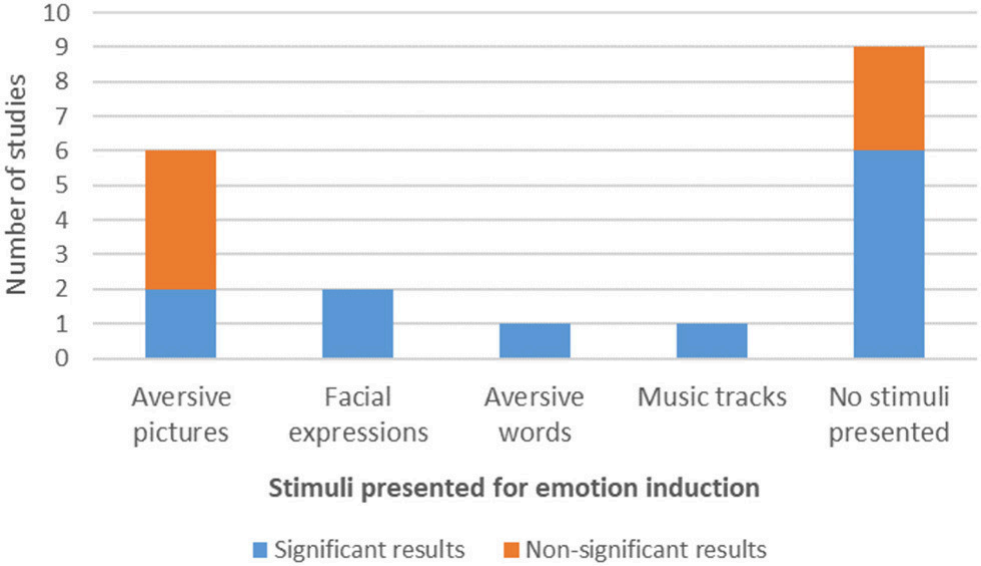
Type of stimuli used for emotion induction during NF protocols and amygdala modulation results.

**Figure 3:**
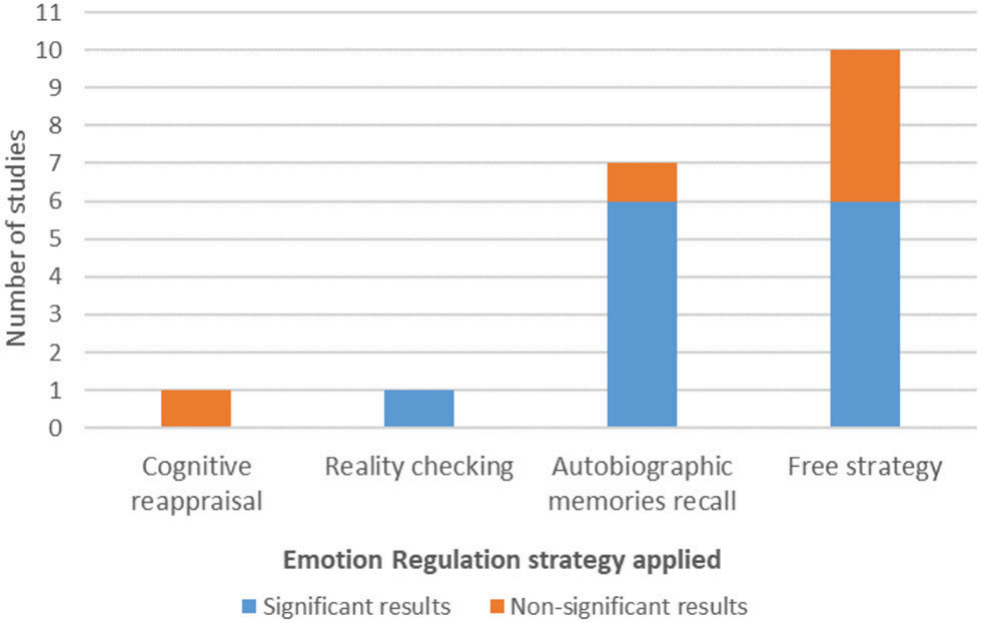
Emotion regulation strategy and amygdala modulation results.

**Figure 4:**
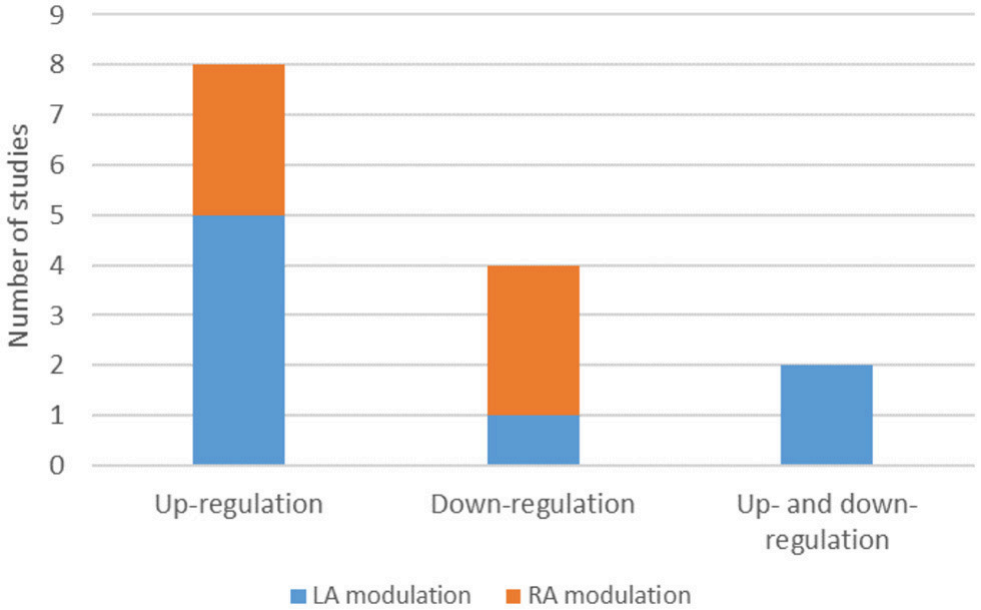
Number of studies on up-regulation, down-regulation, up and down-regulation and amygdala lateralized response. LA, Left amygdala; RA, Right amygdala.

**Figure 5:**
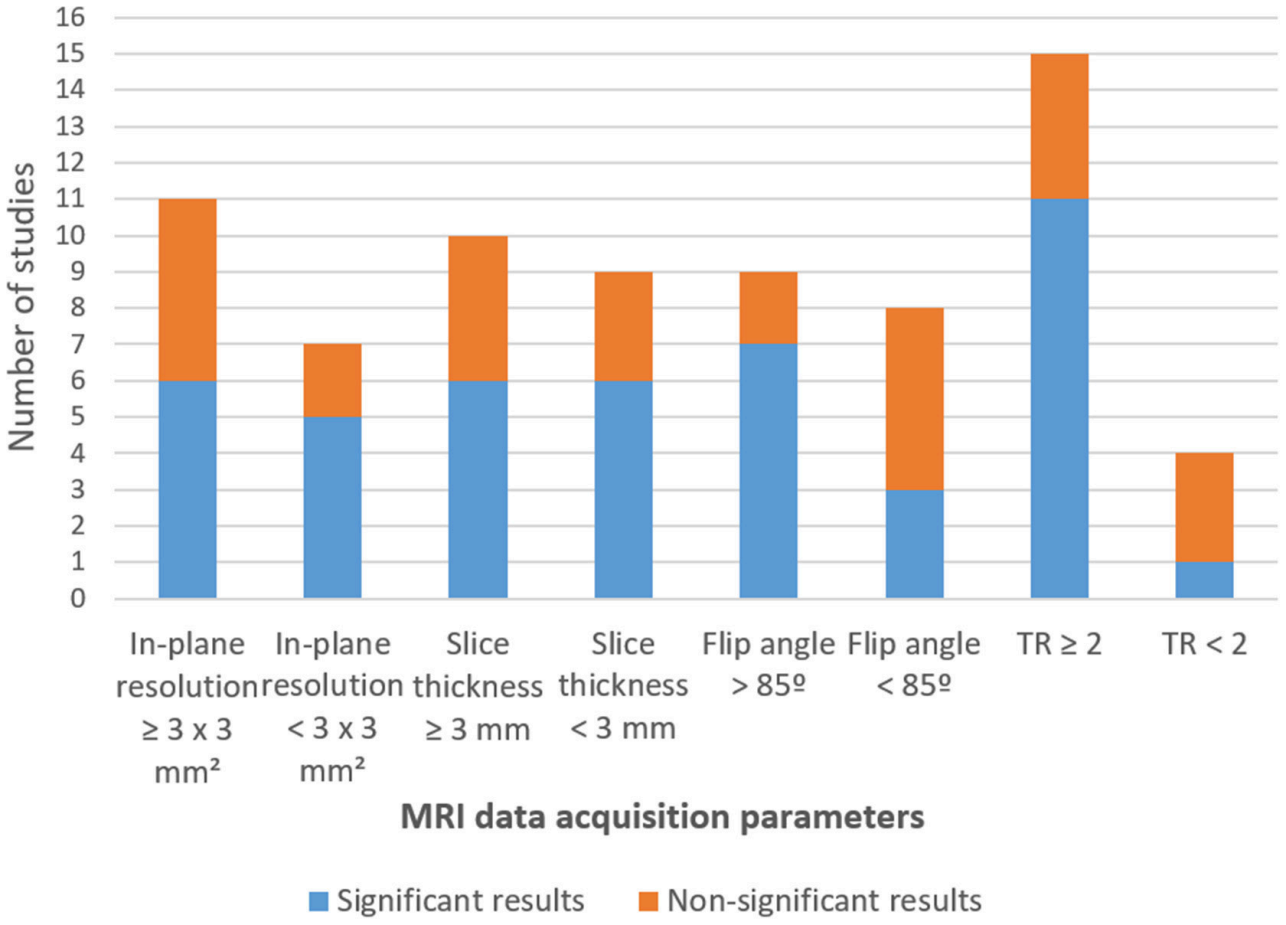
Number of studies by statistical output concerning fMRI data acquisition parameters.

From the final set of papers included, the extraction and categorization of the following detailed features was performed (Tables 1–3a,b) regarding *design and participants*: (1) *study design*, considering two variables: the Up-Regulation/Down-Regulation of amygdala activity; and the existence of a Control Group (whether or not a Control Group was used and if in that group a sham-feedback or no feedback was used). The use of a Control Group does not always refer to sham-NF, as the study may use a Control Group without providing feedback, real or fake—we made this distinction in the reporting of results; (2) *participants' characterization* (sample size, age, gender distribution, exclusion criteria applied for recruitment) and, in particular, the study population—if patients or healthy controls; regarding the *NF protocol*: (3) *instruction* (whether or not there was an ER strategy applied and if there was a previous practice task), (4) *stimuli* (the type of stimuli presented and if stimuli were previously tested for selection), (5) *feedback interface* [type of feedback display—visual, or other type; the feedback source signal—traditional activation studies that used a region-of-interest (ROI) or studies that focused on feedback based on connectivity measures or the classification of distributed patterns of activity, e.g., Multi-Voxel Pattern Analysis (MVPA); in cased where ROIs were used, we specified the feedback ROI]; (6) *protocol design* (the number of NF Training Runs, Practice Run, Transfer Run, and Sessions); (7) *data acquisition parameters* and/or measures [scanner, in-plane voxel resolution, slice thickness, TR, TE that specified single or multi-echo acquisition, flip angle (FA), physiological measurements (heart rate, respiratory rate) and quality control measures] and (8) *data analysis*, that specified online/real-time and offline/*post-hoc* procedures in terms of preprocessing (slice time correction, motion correction, drift/artifacts removal, distortion correction), spatial smoothing (mm), and template (coordinate space), as well as method of statistical analysis used in real time and in post-processing [e.g., General Linear Model (GLM), correlation analysis]. Data concerning the quantitative results were then collected and summarized (Tables 4a,b). Importantly, only direct contrasts (*t*-tests, *Z*-tests) and correlations between Conditions/Runs/Sessions were reported (main or interaction effects were not included in the analysis, with only *post-hoc* tests of these effects being considered). For data visualization, a summary of the results is presented in Figures 2–**5**. Modulation of amygdala activity was considered whenever at least one of the contrasts within studies presented in Table 4a or Table 4b reported significant statistical results.

**Table 1:** Study characteristics and design.

**Study #**	**References**	**Up/down regulation**	**Participants**	**Study design**	**Control group**	***N***	**Age range**	**Gender distribution (F)**	**Participants' exclusion criteria**
1	Brühl et al., 2014	Down-regulation	Healthy	2 conditions: Regulate and View	No	6	22–30	6	1, 3, 4
2	Hellrung et al., 2018	Up-regulation and down-regulation	Healthy	Regulate, Count and Neutral	Yes—No feedback	42	23–30	0	1, 2, 3
3	Johnston et al., 2010	Up-regulation	Healthy	1 condition: Regulate	No	13	21–52	9	1
4	Koush et al., 2015	Top-down connectivity regulation	Healthy	2 conditions: Regulate and Neutral	Yes—Sham NF	15	24–28	8	1, 5
5	Li et al., 2016	Up-regulation	Healthy	1 condition: Regulate	Yes—No NF	23	21–25	5	1, 2, 5
6	Lorenzetti et al., 2018	Up-regulation	Healthy	Regulate, Neutral	No	8	23–28	3	1, 5
7	Marxen et al., 2016	Up-regulation and Down-regulation	Healthy	1 condition: Regulate	No	32	18–40	15	1, 2, 3, 5
8	Nicholson et al., 2017	Down-regulation	PTSD	3 conditions: Regulate, View, Neutral	No	10	43–55	6	1, 3, 4
9	Paret et al., 2014	Down-regulation	Healthy	3 conditions: Regulate, View and Neutral	Yes—Sham NF	32	19–34	32	1
10	Paret et al., 2016	Down-regulation	BPD	3 conditions: Regulate, View, Neutral	No	10	23–43	10	1, 3, 4
11	Paret et al., 2018	Up-regulation and down-regulation	Healthy	View, regulate, and rest	No	20	20–29	20	1
12	Posse et al., 2003a	Up-regulation	Healthy	Regulate	No	6	22–42	4	1, 4
13	Sarkheil et al., 2015	Down-regulation	Healthy	2 conditions: Regulate and View	Yes—No NF	14	20–27	8	1, 2
14	Young et al., 2014	Up-regulation	MDD	3 conditions: Regulate, View and Neutral	Yes—Sham NF	21	18–55	N.a.	1, 3, 4
15	Young et al., 2017	Up-regulation	MDD	Regulate, Count and Neutral	Yes—Sham NF	36	18–55	0	1, 2, 3, 4
16	Zotev et al., 2011	Up-regulation	Healthy	3 conditions: Regulate, Count and Neutral	Yes—Sham NF	28	19–37	0	1, 2, 3, 4
17	Zotev et al., 2014	Up-regulation	Healthy	3 conditions: Regulate, Count, Neutral	No	6	15–33	4	N.a.
18	Zotev et al., 2016	Up-regulation	MDD	3 conditions: Regulate, Count, Neutral	Yes—Sham NF	24	32–50	18	2
19	Zotev et al., 2018	Up-regulation and down-regulation	PTSD	Regulate, Count and Neutral	Yes—Sham NF	23	25–45	0	N.a.

*List of included articles with data extraction. N.a., Information not available; N, sample size; F, number of female participants; Morbidities: PTSD, Post-Traumatic Stress Disorder; MDD, Major Depression Disorder; BPD, Borderline Personality Disorder; Exclusion criteria: (1) History of neurological or psychiatric disease, (2) not right handed, (3) non-compliance with fMRI standards, (4) alcohol or drug abuse, (5) not having normal or corrected-to normal vision*.

**Table 2:** Neurofeedback protocol.

**#**	**References**	**Instruction**	**Emotion regulation strategy training**	**Stimuli**	**Stimuli testing**	**fMRI localizer**	**Feedback display**	**Feedback source**	**Feedback ROI**	**# NF run**	**Duration of regulation trial (s)**	**Number of trials in the regulation block**	**Total duration of regulation block (s)**	**Strategy to prevent amygdala habituation**	**Practice run**	**Transfer run**	**# Sessions**
1	Brühl et al., 2014	Reality checking	Yes	Negative Emotional Faces	No	Yes	Visual	Target ROI activation—% of BOLD signal change	Right amygdala	1	20	10	200	Pictures were randomized and 50% of the pictures were unseen	No	No	4
2	Hellrung et al., 2018	Autobiographic memories recall	Yes	No stimuli presented	No	No(^*^)	Visual	Target ROI activation—% of BOLD signal change	Left amygdala	3	40	4	160	Not reported	Yes	Yes	1
3	Johnston et al., 2010	Free strategy	No	Aversive pictures	Pre-assessment of arousal and valence	Yes	Visual	Target ROI activation—% of BOLD signal change	Bilateral amygdala	3	20	12	240	Not reported	No	No	1
4	Koush et al., 2015	Free strategy	No	Aversive pictures	Pre-assessment of arousal and valence	No	Visual	Effective connectivity value (log Bayes factor in DCM approach)	N.a.	2	12	4	48	The order of presentation was pseudorandomized, and no image was presented more than once to any participant	No	Yes	3
5	Li et al., 2016	Free strategy	Yes	No stimuli presented	No	No	Visual	Emotional Classes (based on MVPA)	N.a.	3	30	6	180	Not reported	No	No	1
6	Lorenzetti et al., 2018	Free strategy	Yes	Music tracks	No	No	Visual	Target ROI activation—% of BOLD signal change AND Support Vector Machine Analysis	Right amygdala	3	46	1	608	Not reported	Yes	No	1
7	Marxen et al., 2016	Free strategy	No	No stimuli presented	No	No	Visual	Target ROI activation—% of BOLD signal change	Bilateral amygdala	3	30.48	4	121.92	Not reported	No	No	3
8	Nicholson et al., 2017	Free strategy	No	Aversive words	No	No	Visual	Target ROI activation—% of BOLD signal change	Bilateral amygdala	3	24	5	120	Not reported	No	Yes	1
9	Paret et al., 2014	Free strategy	No	Aversive pictures	Post-assessment of arousal and valence	No	Visual	Target ROI activation—% of BOLD signal change	Bilateral amygdala	3	24	5	120	Not reported	No	Yes	1
10	Paret et al., 2016	Free strategy	No	Aversive pictures	Post-assessment of arousal and valence	No	Visual	Target ROI activation—% of BOLD signal change	N.a.	3	24	5	120	Not reported	No	Yes	4
11	Paret et al., 2018	Free strategy	No	Aversive pictures	Post-assessment of arousal and valence	No	Visual	Target ROI activation—% of BOLD signal change	Right amygdala	5	18	5	54	Not reported	No	No	1
12	Posse et al., 2003a	Free strategy	No	Negative emotional faces	No	No	Auditory	Target ROI activation—% of BOLD signal change	Bilateral amygdala	1	30	240	7,200	Not reported	No	No	1
13	Sarkheil et al., 2015	Cognitive reappraisal	Yes	Aversive pictures	Pre-assessment of arousal and valence	Yes	N.a.	Target ROI activation—% of BOLD signal change	Lateral Prefrontal Cortex	3	4.5	3	13.5	Not reported	No	No	1
14	Young et al., 2014	Autobiographic memories recall	Yes	No stimuli presented	No	No	Visual	Target ROI activation—% of BOLD signal change	Left amygdala	3	40	4	160	Not reported	Yes	Yes	1
15	Young et al., 2017	Autobiographic memories recall	No	No stimuli presented	No	No	Visual	Target ROI activation—% of BOLD signal change	Left amygdala	3	40	4	160	Not reported	Yes	Yes	2
16	Zotev et al., 2011	Autobiographic memories recall	Yes	No stimuli presented	No	No	Visual	Target ROI activation—% of BOLD signal change	Left amygdala	3	40	4	160	Not reported	Yes	Yes	1
17	Zotev et al., 2014	Autobiographic memories recall	Yes	No stimuli presented	No	No	Visual	Target ROI activation—% of BOLD signal change	Left amygdala	3	40	4	160	Not reported	Yes	Yes	1
18	Zotev et al., 2016	Autobiographic memories recall	Yes	No stimuli presented	No	No	Visual	Target ROI activation—% of BOLD signal change	Left amygdala	3	40	4	160	Not reported	Yes	Yes	1
19	Zotev et al., 2018	Autobiographic memories recall	Yes	No stimuli presented	No	No	Visual	Target ROI activation—% of BOLD signal change	Left amygdala	3	40	4	160	Not reported	Yes	Yes	3

Data extraction about protocol features. BOLD, Blood-oxygen-level-dependent contrast imaging; DCM, dynamic causal modeling; MVPA, multi-voxel pattern analysis; N.a., Information not available; NF, Neurofeedback; s, seconds. (^*^)A short localizer run (20 s) at the beginning of the experiment was applied for amygdala mask positioning (participants were instructed to relax).

**Table 3a:** Data acquisition parameters.

**Study #**	**References**	**fMRI data acquisition parameters**								**Quality control measures**	**Physiological measurements**	

		**Scanner**	**In-plane resolution (mm**^**2**^**)**	**Slice thickness (mm)**	**Flip angle**	**Parallel imaging reconstruction techniques**	**Repetition Time (TR) (ms)**	**Echo time (TE) (ms)**	**Section orientation**		**Heart rate monitoring**	**Respiratory rate monitoring**
1	Brühl et al., 2014	3T	3 × 3	3	N.a.	SENSE R = 2	2,000	25	axial	Verbal questioning; checked for excessive head movements	No	No
2	Hellrung et al., 2018	3T	3 × 3	2.6	90°	N.a.	2,000	25	axial (AC-PC orientation)	No	No	No
3	Johnston et al., 2010	3T	2 × 2	3	N.a.	N.a.	2,000	30	N.a.	N.a.	N.a.	N.a.
4	Koush et al., 2015	3T	1.8 × 1.8	1.8	70°	GRAPPA, iPAT = 3	1,100	30	N.a.	N.a.	Yes	Yes
5	Li et al., 2016	3T	3.4375 × 3.4375 (^*^)	3.5	80°	N.a.	2,000	30	N.a.	Checked for excessive head movements	N.a.	N.a.
6	Lorenzetti et al., 2018	3T	3.75 × 3.75	3.75	90°	SENSE R = 1.5	2,000	22	N.a.	Self-reports of tiredness and focus	No	No
7	Marxen et al., 2016	3T	4 × 4	3.2	82°	GRAPPA, iPAT = 3	2,540	8.6, 18.3, 28, 38, 48, 57^a^	near-axial (axial tilted toward coronal)	N.a.	Yes	Yes
8	Nicholson et al., 2017	3T	3 × 3	3	80°	N.a.	2,000	30	near-axial (slices tilted–20° from AC-PC orientation	Participants'heads were stabilized	N.a.	N.a.
9	Paret et al., 2014	3T	N.a. (^**^)	3	80°	N.a.	2,000	30	axial (AC-PC orientation)	Eye-Tracking	N.a.	N.a.
10	Paret et al., 2016	3T	3 × 3	3	80°	N.a.	2,000	30	near-axial (slices tilted–20° from AC-PC orientation)	Eye-Tracking	N.a.	N.a.
11	Paret et al., 2018	3T	3 × 3	3	60°	GRAPPA	1,000	30	near-axial (slices tilted−20° from AC-PC orientation)	Eye-Tracking	No	No
12	Posse et al., 2003a	1.5T	6.25 × 6.25	3	30°	N.a.	1,000	12–140^a^	axial or near-axial (AC-PC orientation)	No	Yes	Yes
13	Sarkheil et al., 2015	3T	3 × 3	2.5	90°	N.a.	1,500	30	axial	N.a.	N.a.	N.a.
14	Young et al., 2014	3T	1.875 × 1.875	2.9	90°	SENSE R = 2	2,000	30	axial	N.a.	Yes	Yes
15	Young et al., 2017	3T	1.875 × 1.875	2.9	90°	SENSE R = 2	2,000	30	axial	No	Yes	Yes
16	Zotev et al., 2011	3T	1.875 × 1.875	2.9	90°	SENSE R = 2	2,000	30	axial	N.a.	Yes	Yes
17	Zotev et al., 2014	3T	3.75 × 3.75	2.9	90°	SENSE R = 2	2,000	30	axial	N.a.	Yes	Yes
18	Zotev et al., 2016	3T	1.875 × 1.875	2.9	90°	SENSE R = 2	2,000	30	axial	N.a.	Yes	Yes
19	Zotev et al., 2018	3T	1.875 × 1.875	2.9	90°	SENSE R = 2	2,000	30	axial	No	Yes	Yes

*Information regarding fMRI data acquisition*.

a The study used multi-echo acquisition to optimize BOLD sequence; AC, anterior commissure; N.a., No information available; N.c., Not clear; SENSE, Sensitivity Encoding (SENSE acceleration factor)—enhancing the performance of magnetic resonance imaging (MRI) by means of arrays of multiple receiver coils through sensitivity encoding (Pruessmann et al., 1999); R, acceleration factor (or reduction factor); GRAPPA, generalized autocalibrating partially parallel acquisitions; iPAT, Integrated Parallel Acquisition Techniques (the iPAT number refers to the image acceleration factor or reduction in the length of the echo train); PC, posterior commissure; (^*^) in-plane resolution was computed using the formula Field-of-View/Acquisition Matrix; (^**^) only Field-of-View is reported: 192 × 192, not acquisition matrix nor direct in-plane resolution.

**Table 3b:** Online and offline data processing.

		**Online/real-time processing**				**Offline/*post-hoc* processing**			
**Study #**	**References**	**ROI definition for online extraction of BOLD signal**	**ROI sphere diameter (mm)**	**Preprocessing**	**Spatial smoothing (mm)**	**Offline extraction of BOLD signal**	**Preprocessing**	**Spatial smoothing (mm)**	**Template**
1	Brühl et al., 2014	Functionally defined using pictures^*^	9	2, 3	N.a.	Event-related averaging of ROI	1, 2, 3	4	Talairach
2	Hellrung et al., 2018	Mask through anatomical parcellation	N.a.	2	N.a.	Percent signal changes related to baseline	2, 4	8	MNI
3	Johnston et al., 2010	Functionally defined using pictures^*^	N.a.	2	N.a.	GLM calculation	2, 3	4	Talairach
4	Koush et al., 2015	Coordinates^***^	N.a.	2, 3	N.a.	Bayesian model comparison between the 2 model alternatives using DCM10 (including both baseline and regulation condition)	2, 3, 4	5	Talairach
5	Li et al., 2016	Anatomical mask^**^	7	2	Yes	z-scoring	1, 2	6	MNI
6	Lorenzetti et al., 2018	Anatomical mask ** (ROI method) and *** (SVM method)	N.a.	2	N.a.	Percent signal changes related to baseline	1, 2, 3 (4)	N.a.	MNI
7	Marxen et al., 2016	Mask through anatomical parcellation	N.a.	2, 4	N.a.	Percent signal changes related to baseline (rest condition)	1, 2, 3	8	N.a.
8	Nicholson et al., 2017	Anatomical mask^**^	15	2	4	Beta-values discrimination	1, 2	6	MNI
9	Paret et al., 2014	Anatomical template (brain atlas)^**^	8	2	4	Beta-values discrimination	1, 2	6	MNI
10	Paret et al., 2016	Mask through anatomical parcellation^**^	20	2	4	Percent signal changes related to baseline (rest condition)	1, 2, 3, 4	8	MNI
11	Paret et al., 2018	Anatomical template (brain atlas)	N.a.	2, 3	N.a.	Percent signal changes related to baseline	2, 4	8	MNI
12	Posse et al., 2003a	Anatomical mask	N.a.	2	N.a.	Cumulative correlation analysis	2	N.a.	N.a.
13	Sarkheil et al., 2015	Anatomical mask^**^	5	2, 3	N.a.	Percent signal changes related to baseline (rest condition) – with an adjustment (x50)	1, 2, 3	6	Talairach
14	Young et al., 2014	Coordinates^***^	14	2	N.a.	Percent signal changes related to baseline (rest condition)	1, 2	5	Talairach
15	Young et al., 2017	Coordinates^***^	14	2	N.a.	Percent signal changes related to baseline	N.a.	N.a.	Talairach
16	Zotev et al., 2011	Coordinates^***^	14	2	N.a.	Percent signal changes related to baseline (rest condition)	1, 2	5	Talairach
17	Zotev et al., 2014	Coordinates^***^	14	2	N.a.	Percent signal changes related to baseline (rest condition)	1, 2	6	Talairach
18	Zotev et al., 2016	Coordinates^***^	14	2	N.a.	Percent signal changes related to baseline (rest condition)	1, 2, 3	5	Talairach
19	Zotev et al., 2018	Coordinates^***^	14	2	N.a.	Percent signal changes related to baseline	1, 2, 3	N.a.	Talairach

N.a., No information available, MNI, Montreal Neurological Institute coordinates space; SVM, Support Vector Machine; Preprocessing: 1. Slice timing correction, 2. Head motion correction, 3. Drift/artifact removal, 4. Distortion correction; mm, millimeters;

* Pictures from an international validated system considering high ratings in arousal and valence to identify the brain emotion-responsive area;

** An anatomical mask is applied, selecting the highest beta-values between the Regulate Condition and the Neutral Condition;

*** *based on previous functional neuroimaging studies*.

**Table 4a:** Statistical results of contrasts to test amygdala modulation (I)—Training effects.

			**Selected data**						
			**Experimental group**					**Control Group**	
			**Last run**	**Transfer run**	**First run**	**Regulate condition**		**Last run**	**Transfer run**
			**Contrast**						
**Study #**	**References**		**Regulate condition vs. control condition**	**Regulate condition vs. control condition**	**Regulate condition vs. control condition**	**Transfer run vs. last run**	**First run vs. last run**	**Regulate condition** **vs. control** **condition**	**Regulate condition** **vs. control** **condition**
1	Brühl et al., 2014	RA					***t***_**(5)**_ **=-4.924**, ***p*** **=** **0.004**		
2	Hellrung et al., 2018	LA					**Δ** **=** **0.57**		
3	Johnston et al., 2010				***t***_**(12)**_ **=** **3.98**, ***p*** **=** **0.002**		***t***_**(12)**_ **=** **2.46**, ***p*** **=** **0.029**		
4	Koush et al., 2015^a^, ^b^								
5	Li et al., 2016^a^	LA					*t*_(9)_ = 1.20, **p** = 0.13		
6	Lorenzetti et al., 2018	RA	***t*** **=** **6.98**, ***p*** **<** **0.05**						
7	Marxen et al., 2016			*d* = 0.43		*R* = 0.542, *p* = 0.001			
8	Nicholson et al., 2017^b^	RA		***t***_**(11)**_ **=** **−3.64**, ***p*** **=** **0.001**					
		LA		***t***_**(11)**_ **=** **−2.18**, ***p*** **<** **0.025**					
9	Paret et al., 2014^a^, ^b^	RA		***t***_**(15)**_ **=** **2.797**, ***p*** **=** **0.007**					*t*_(14)_ = 0.451, *p* = 0.330
		LA		*t*_(15)_ = 0.178, *p* = 0.861					*t*_(15)_ = 0.099, *p* = 0.923
10	Paret et al., 2016^b^			*t*_(7)_ < 2.0, *p* > 0.1					
11	Paret et al., 2018	RA							
12	Posse et al., 2003a	RA^*^			***t***_**(6)**_ **=** **3.4**, ***p*** **<** **0.05**				
		LA^*^			***t***_**(6)**_ **=** **7.4**, ***p*** **<** **0.05**				
		RA^**^			***t***_**(6)**_ **=** **3.4**, ***p*** **<** **0.05**				
		LA^**^			***t***_**(6)**_ **=** **4.6**, ***p*** **<** **0.05**				
13	Sarkheil et al., 2015	LA					*t*_(7)_ = 0.90, *p* = 0.30		
		RA					*t*_(7)_ = 1.56, *p* = 0.16		
14	Young et al., 2014^a^, ^b^	LA				***t***_**(13)**_ **=** **0.34**, ***p*** **=** **0.37**			
15	Young et al., 2017	LA				*t* > 2.34, *p* < 0.03			
16	Zotev et al., 2011^a^, ^b^	LA				***t***_**(13)**_ **=** **0.01**, ***p*** **=** **0.992**			
		RA				***t***_**(13)**_ **=** **0.31**, ***p*** **=** **0.765**			
17	Zotev et al., 2014^b^	LA		***t***_**(5)**_ **=** **4.51**, ***p*** **<** **0.006**					
18	Zotev et al., 2016^a^, ^b^	LA	*t*_(12)_ = 2.62, *p* < 0.022, *q* < 0.055	***t***_**(12)**_ **=** **4.64**, ***p*** **<** **0.001**		***t***_**(12)**_ **=** **1.22**, ***p*** **<** **0.246**		*t*_(10)_ = 2.32, *p* < 0.043, *q* < 0.118	*t*_(10)_ = 2.26, *p* < 0.047, *q* < 0.118
19	Zotev et al., 2018	LA	***t***_**(18)**_ **=** **3.42**, ***p*** **<** **0.003**	***t***_**(18)**_ **=** **2.52**, ***p*** **<** **0.021**		***t***_**(18)**_ **=** **1.66**, ***p*** **<** **0.114**			

Bold features highlight significant results (note: for the contrast “TR vs. LR” highlighted results are the non-significant ones, given that no differences between the Last Training Run and the Transfer Run corroborates that amygdala modulation is maintained even without feedback);

a The study included a control group;

b the study included a Transfer Run; LR, Last Run; FR, First Run; TR, Transfer Run; RC, Regulate Condition; CC, Control Condition; RA, Right Amygdala; LA, Left Amygdala;

* *male faces*,

** *female faces*.

**Table 4b:** Statistical results of contrasts to test amygdala modulation (I)—Group effects.

			**Selected data**	
			**Last run**	**Transfer run**
			**Contrast**	
**Study #**	**References**		**Experimental group vs. control group**	**Experimental group vs**. **control group**
1	Brühl et al., 2014			
2	Hellrung et al., 2018			***F***_**(2, 24)**_ **=** **4.53**, ***p*** **=** **0.04**
3	Johnston et al., 2010			
4	Koush et al., 2015^a^, ^b^			
5	Li et al., 2016^a^		*t*_(19)_ = 1.15, *p* = 0.27	
6	Lorenzetti et al., 2018			
7	Marxen et al., 2016			
8	Nicholson et al., 2017^b^			
9	Paret et al., 2014^a^, ^b^			
10	Paret et al., 2016^b^			
11	Paret et al., 2018			
12	Posse et al., 2003a			
13	Sarkheil et al., 2015^a^			
14	Young et al., 2014^a^, ^b^	RA	***t***_**(19)**_ **=** **2.60**, ***p*** **=** **0.01**	***t***_**(19)**_ **=** **2.02**, ***p*** **=** **0.035**
15	Young et al., 2017			
16	Zotev et al., 2011^a^, ^b^	LA	***t***_**(26)**_ **=** **2.70**, ***p*** **=** **0.012**	***t***_**(26)**_ **=** **2.47**, ***p*** **=** **0.020**
		RA	*t*_(26)_ = 1.83, *p* = 0.079	*t*_(26)_ = 1.63, *p* = 0.115
17	Zotev et al., 2014^b^			
18	Zotev et al., 2016^a^, ^b^			
19	Zotev et al., 2018		*t*_(28)_ = 1.84, *p* < 0.076	

a The study included a control group;

b *The study included a Transfer Run. Bold features highlight significant results; RA, Right Amygdala; LA, Left Amygdala*.

#### Data Analysis

Although the original idea of the systematic review was to gather data to perform a quantitative meta-analysis, the heterogeneity of study methods and reporting precluded this type of analysis. Therefore, a qualitative review of the studies satisfying inclusion criteria was performed instead.

We reviewed studies regarding the amygdala response to the NF training and the differences in the methodology used in these studies, by extracting and summarizing (a) Study characteristics—study design, neurofeedback protocol features, and data acquisition parameters, and (b) Amygdala modulation—how data acquisition parameters, study design, and neurofeedback protocol features relate to results. The following characteristics and points were analyzed: Exclusion criteria, Single vs. Multiple Sessions, Experimental vs. Control Group, rtfMRI-NF protocol design, Transfer Run and Practice Run, Stimuli, Emotion Regulation Strategies, Data acquisition parameters, and Amygdala lateralization.

## Results

### Literature Search

The review of the literature using search items as described above identified 531 potential target articles (84 identified via the PUBMED database, 323 through Science Direct and 117 via Web of Science).

In the first stage of selection, 484 papers were excluded (Figure 1), following the previously defined inclusion criteria (1) to (6) (section Eligibility Criteria, Screening Phase, and Study Selection): 122 were duplicated records, 191 were reviews or non-original research articles, 111 were not rtfMRI-NF based studies, 55 did not address the training of ER processes, 20 did not make direct and separate measurements in the amygdala, and two were not written in English. Thus, 46 articles were selected for full text analysis. After carefully evaluating all the experimental paradigms used, in a second selection stage we excluded 27 more articles for the following reasons: three studies consisted of a new analysis approach made to the same experimental paradigm previously described in another paper already included in our pool, showing no new quantitative results for the BOLD activity in amygdala during NF; 15 showed no quantitative results regarding modulation of amygdala's activity as a training effect; five did not use the BOLD signal as the NF source in the protocol; and the remaining four dealt with “EEG Finger-Print” analysis-based NF, with pre and post-training fMRI data but featured an EEG-based NF protocol.

Finally, 19 articles were selected for further quantitative analysis. These referred to 19 studies that used rtfMRI-NF protocols to train ER and presented quantitative results regarding amygdala responses. The characterization of studies, NF protocols and data acquisition parameters are described in detail in the Tables and summarized in the Figures.

### Feasibility of Quantitative Analysis

Efforts were made in order to find a common and comparable statistical outcome measure to attempt a meta-analysis of the main effects. However, the large heterogeneity of analysis approaches found in the set of reports precluded such strategy. A large array of different statistical outcome measures among studies were found, reporting the effects of NF training based on 23 different variable contrasts that were reported (by outcome measure we refer here to the specific contrasts between the variables or conditions employed by the authors to measure the effect of ER in amygdala BOLD signal). A selection of the most representative outcome measures is reported in Tables 4a,b. Because of the variability in the statistical measurements of amygdala modulation, requisites for a valid meta-analysis were not present. Therefore, a non-quantitative analysis of the results and protocol features was performed.

### Non-quantitative Analysis

The next subsections will focus on the qualitative analysis performed regarding the selected descriptive features of the included studies, NF protocols and reported results. First, the study characteristics and rtfMRI-NF protocol variations will be described in the subsection “*Study characteristics—study design, neurofeedback protocol features and data acquisition parameters*.” Second, considering these features, a comparative analysis of the contrasts testing modulation of amygdala activity will be described in the subsection “*Amygdala modulation—how data acquisition parameters, study design, and neurofeedback protocol features relate with results*.”

### Study Characteristics—Participants, Study Design, Neurofeedback Protocol Features, and Data Acquisition Parameters

#### Participants

Among the selected studies, 13 (68.42%) were neuroscience studies (Posse et al., 2003a; Johnston et al., 2010; Zotev et al., 2011, 2014; Brühl et al., 2014; Paret et al., 2014, 2018; Koush et al., 2015; Sarkheil et al., 2015; Li et al., 2016; Marxen et al., 2016; Hellrung et al., 2018; Lorenzetti et al., 2018), and six (31.58%) are clinically focused (Young et al., 2014, 2017; Paret et al., 2016; Zotev et al., 2016, 2018; Nicholson et al., 2017). Two studies addressed Post-Traumatic Stress Disorder (Nicholson et al., 2017; Zotev et al., 2018), one study focused on Borderline Personality Disorder (Paret et al., 2016) and three studies described a sample of subjects diagnosed with Major Depressive Disorder (Young et al., 2014, 2017; Zotev et al., 2016).

In terms of sample characteristics, the age ranges varied between 18 and 55. Sample size ranged from 6 to 42 subjects. Gender distribution was uneven: four studies presented a sample with females exclusively (Brühl et al., 2014; Paret et al., 2014, 2016, 2018) and four studies considered only males (Zotev et al., 2011, 2018; Young et al., 2017; Hellrung et al., 2018).

#### Exclusion Criteria

Concerning the selection of participants, often, non-overlapping exclusion criteria for participants were applied among studies: (a) History of neurological or psychiatric disease, (b) not being right-handed (handedness is taken as a rough measure of hemispheric dominance to ensure sample homogeneity, and reduce confounds), (c) non-compliance with fMRI standards, such as “general contraindications against MRI examinations” (Zotev et al., 2011; Brühl et al., 2014), “general MRI exclusions/incompatibilities” (Young et al., 2014; Paret et al., 2016), which related to the “MRI safety standards” (Nicholson et al., 2017), and “physical conditions that prevent lying comfortably inside an MRI scanner” (Marxen et al., 2016); (d) alcohol/drug abuse, and (e) absence of normal or corrected-to-normal vision. Nine of the studies (47.37%) used three or more of these criteria (Zotev et al., 2011; Brühl et al., 2014; Young et al., 2014, 2017; Li et al., 2016; Marxen et al., 2016; Paret et al., 2016; Nicholson et al., 2017; Hellrung et al., 2018), four studies (21.05%) used two of the mentioned criteria (Posse et al., 2003a; Koush et al., 2015; Sarkheil et al., 2015; Lorenzetti et al., 2018), four studies (21.05%) used only one exclusion criteria (Johnston et al., 2010; Paret et al., 2014, 2018; Zotev et al., 2016) and two did not describe the applied exclusion criteria (Zotev et al., 2014, 2018). From these criteria, (a) was the most frequently used (considered in 16 of the studies−84.21%), secondly (c) (eight studies−42.11%), followed by (d) and (b) (seven studies each−36.84%), and finally (e) being the least used criterion (only four studies−21.05%).

#### Single vs. Multiple Sessions

Only six studies (31.58%) had more than one NF training Session, three studies used three Sessions (Koush et al., 2015; Marxen et al., 2016; Zotev et al., 2018) and two studies performed four Sessions (Brühl et al., 2014; Paret et al., 2016).

#### Experimental vs. Control Group

In the study design, in nine of the reports (47.37%), authors decided not to use a Control Group (Posse et al., 2003a; Johnston et al., 2010; Brühl et al., 2014; Paret et al., 2014, 2018; Zotev et al., 2014; Marxen et al., 2016; Nicholson et al., 2017; Lorenzetti et al., 2018), against 11 (57.89%) who did. Seven (36.84%) used a Control Group with a sham-NF approach (Paret et al., 2014; Young et al., 2014, 2017; Zotev et al., 2014, 2016, 2018; Koush et al., 2015) and three (15.79%) gave no feedback to the participants of the Control Group (Sarkheil et al., 2015; Li et al., 2016; Hellrung et al., 2018). As for the selection of participants' study arm, the great majority of the studies did not describe their approach—only six studies (31.58%) reported a blind and randomized selection process (Zotev et al., 2011; Paret et al., 2014; Koush et al., 2015; Li et al., 2016; Young et al., 2017;Hellrung et al., 2018).

#### fMRI Protocol Design

Most studies applied a block design for the ER task, alternating regulate and rest blocks. In this type of design, volunteers were required to regulate their BOLD signal for one period (Regulate Condition) followed by a rest block. In three study cases (15.79%) rest blocks consisted of presenting emotional stimuli to the participants, without any instruction regarding regulation (View Condition). In 12 studies (63.16%), apart from the View condition, the protocol also included a third block in which the participants were either asked to perform a counting task or were exposed to neutral stimuli with no emotional content (Neutral Condition) or to a Rest block (without any task to perform). For data analysis purposes in this review, Rest blocks (View, Neutral, or Rest Conditions) were considered overall as the “Control Condition”. The Control Condition blocks are generally used to obtain a baseline of the BOLD activity. Baseline values are subsequently used as a reference for the detection of BOLD signal changes, by comparing it with the signal measured during the Regulate Condition. In terms of trial duration on the Regulation blocks (which included the Regulate Condition), variations occurred between 4.5 and 46 s of stimulus display. However, in most of the protocols (78.95%), the Regulate trial lasted between 20 and 46 s and in only 15.79% of the cases the trial lasted <20 s (Koush et al., 2015; Sarkheil et al., 2015; Paret et al., 2018). The number of trials in a Regulation block varied from three to 12 trials per block, with one study having 240 trials in the same (and only) experimental block (Posse et al., 2003a).

Only two articles mentioned considering amygdala habituation effects and described strategies to prevent it: (1) the pictures were randomized and 50% of the pictures were unseen prior to the study (Brühl et al., 2014), and (2) the order of presentation was pseudorandomized, with no image being presented more than once to the participants (Koush et al., 2015).

Five studies (26.32%) were about amygdala Down-Regulation, 9 (47.37%) about amygdala Up-Regulation, four studies attempted both Up- and Down-Regulation of amygdala activity and one study investigated top-down connectivity regulation between amygdala and other regions of the ER neural circuitry (see Table 1).

Feedback sources also differed across studies. Seventeen of the 19 studies (89.47%) assessed the % of BOLD signal change in a specific target ROI as the feedback source, while two other studies used distinct sources: in one study participants' emotional states (positive or negative) were given to them as feedback but with these states being classified using MVPA—Multi-Voxel Pattern Analysis of functional imaging data (Li et al., 2016), whereas a second study used a connectivity feedback approach (Koush et al., 2015). In the study of Li et al. (2016), MVPA provided a binary classification (positive or negative emotion) based on the decoding of distributed brain signals across multiple voxels. Such MVPA classification was then used as feedback for the participants. MVPA investigates the information contained in distributed patterns of neural activity and it is considered as a supervised classification method where a classifier attempts to capture the relationships between spatial pattern of fMRI activity and experimental conditions (in this case positive or negative emotions).

In the study of Koush et al. (2015), the goal of the NF training was to strengthen the top-down connectivity from the dorsolateral Prefrontal Cortex (dmPFC) onto the bilateral amygdala. The connectivity-based NF signal was calculated using a real-time Dynamic Causal Modeling (DCM) approach based on the time courses of three ROIs (dmPFC, left and right amygdala). DCM is a Bayesian framework for modeling effective brain connectivity. The feedback display consisted of a logarithmic Bayes factor value (which was red if the trial was successful, i.e., positive, and blue otherwise). This value included the cumulative reward that had been earned until then. During the NF training, the participants learned to voluntarily increase top-down effective connectivity from the dmPFC onto the bilateral amygdala. This was accomplished by providing a feedback signal that indicated the degree of dominance of a top-down model (target model for training) compared with a bottom-up model. If the top-down model fit the ongoing brain activity during a training trial better than the bottom-up model, the feedback signal was positive; if the bottom-up model dominated, the feedback signal was negative. NF training was therefore focused on up-regulating positive emotions. In other words, if the logarithmic Bayes factor was positive, the participant was rewarded for a successful trial. Importantly, the feedback signal calculation for a NF trial was based on the entire ROI time series of this trial, including baseline and Regulate Conditions. After each repetition of the five baseline and the four Regulation blocks, participants were given the chance to rest for 38 s. Afterwards, participants were presented with feedback about their success for 4 s. Feedback was therefore intermittent and slow.

From the 17 studies that defined a specific brain region as the feedback target, and not a connectivity approach (89.47%), five studies (29.41%) defined bilateral amygdala (Johnston et al., 2010; Paret et al., 2014, 2018; Marxen et al., 2016; Nicholson et al., 2017) as the feedback ROI; seven studies (41.18%) used only the left amygdala (Zotev et al., 2011, 2014, 2016, 2018; Young et al., 2014, 2017; Hellrung et al., 2018), whereas three studies used only the right amygdala (Brühl et al., 2014; Lorenzetti et al., 2018; Paret et al., 2018). One study defined another brain region as the target ROI (lateral PFC) while analyzing modulation in the amygdala (Sarkheil et al., 2015).

#### Transfer Run and Practice Run

Similarly, there was a lot of variability concerning the NF protocol features. Eleven studies (57.89%) included a Transfer Run in the protocol design (Zotev et al., 2011, 2014, 2016, 2018; Paret et al., 2014, 2016; Young et al., 2014, 2017; Koush et al., 2015; Nicholson et al., 2017; Hellrung et al., 2018) to determine whether or not the participants were able to demonstrate enhanced brain activation or other behavioral effects when feedback is no longer available. Regarding the Practice Run, only eight studies (42.11%) included a run in which the participants could practice the regulation strategy task without receiving any kind of feedback, before the NF runs (Zotev et al., 2011, 2014, 2016, 2018; Young et al., 2014, 2017; Hellrung et al., 2018; Lorenzetti et al., 2018).

#### Stimuli

Regarding the stimuli, 9 studies did not present any kind of stimuli to their participants to induce an emotional state, eight studies (42.11%) used picture stimuli, one study used (aversive) words (Nicholson et al., 2017) and one other study used music tracks (Lorenzetti et al., 2018). Six (31.57%) of the studies employing pictures used aversive pictures (Johnston et al., 2010; Paret et al., 2014, 2016, 2018; Koush et al., 2015; Sarkheil et al., 2015) and the two other studies used negative emotional faces of the same gender and emotional valence (Posse et al., 2003a; Brühl et al., 2014).

Of the studies employing stimuli, seven studies (70%) did not report performing any type of previous stimuli assessment in terms of emotion induction potential, whereas three studies described a pre-assessment of arousal and valence of the stimuli (before the experiment) (Johnston et al., 2010; Koush et al., 2015; Sarkheil et al., 2015). In two of the cases the assessment was made with the same participants as the main task. Finally, three studies reported a post-assessment of arousal and valence of the stimuli (after training), with the same participants performing the NF main task (Paret et al., 2014, 2016, 2018).

#### Localizer Run

Only three studies reported the use of a functional localizer run to define the NF target ROI online (Johnston et al., 2010; Brühl et al., 2014; Sarkheil et al., 2015).

#### Emotion Regulation Strategies

Concerning the application of ER strategies, not all of the studies explicitly instructed their participants to use a defined ER strategy. Indeed, in 10 of the studies (52.63%) the authors told the participants to use a free ER strategy, meaning that no defined strategy was required (Posse et al., 2003a; Johnston et al., 2010; Paret et al., 2014, 2016, 2018; Koush et al., 2015; Li et al., 2016; Marxen et al., 2016; Nicholson et al., 2017; Lorenzetti et al., 2018). Only two studies (10.53%) considered the use of cognitive ER strategies: Cognitive Reappraisal (Sarkheil et al., 2015) and Reality Checking (Brühl et al., 2014). The other seven studies (36.84%) instructed participants to Recall Autobiographic Memories (Zotev et al., 2011, 2014, 2016, 2018; Young et al., 2014, 2017; Hellrung et al., 2018).

#### Data Acquisition Parameters

Regarding the data acquisition parameters, the majority of studies (94.74%) were conducted using 3T MRI equipment. Respecting EPI parameters, in-plane resolution ranged between 1.8 × 1.8 and 6.25 × 6.25 mm^2^, and slice thickness between 1.8 and 3.75 mm. Relatively low variability was found in TR (14 studies with TR = 2,000 ms, one study with TR > 2,000 ms and four studies with TR < 2,000 ms) and TE (from the 19 studies that reported this information, 14 defined a TE = 30 ms, and three studies defined TE ≤ 25 ms) values. Only two studies (Posse et al., 2003a; Marxen et al., 2016) used multi-echo acquisition to improve BOLD sensitivity using a TE range of 8.6–57 ms and of 12–140 ms, respectively.

All the studies used axial or near-axial slice orientation except for four studies, in which this information was lacking (Johnston et al., 2010; Koush et al., 2015; Li et al., 2016; Lorenzetti et al., 2018). Of the 17 studies that reported FA, nine studies used a 90° angle, whereas the other eight reported FAs equal or inferior to 82° (Table 3a).

Of the 19 studies included, 11 employed parallel imaging acceleration for image reconstruction. Three studies used GRAPPA (Koush et al., 2015; Marxen et al., 2016; Paret et al., 2018); and the other eight studies used SENSE (Zotev et al., 2011, 2014, 2016, 2018; Brühl et al., 2014; Young et al., 2014, 2017; Lorenzetti et al., 2018). Of the studies employing parallel imaging, only one used this technique combined with multi-echo acquisition (Marxen et al., 2016).

#### Online and Offline Data Processing

Different online data processing steps and parameters were reported across the reviewed studies. For ROI definition, eight studies considered the coordinates given by previous functional neuroimaging studies (Zotev et al., 2011, 2014, 2016, 2018; Young et al., 2014, 2017; Koush et al., 2015; Sarkheil et al., 2015), four studies applied an anatomical mask (Li et al., 2016; Nicholson et al., 2017; Hellrung et al., 2018; Paret et al., 2018), two studies functionally defined the ROI using pictures from an international validated system (Johnston et al., 2010; Brühl et al., 2014), and one study used a mask obtained through anatomical parcellation (Marxen et al., 2016). The ROI sphere diameter varied across studies from 5 to 20 mm, although seven studies did not report this parameter. Four of the studies that reported ROI size referred a diameter between 5 and 9 mm, and eight studies reported a diameter larger than 10 mm (Zotev et al., 2011, 2014, 2016, 2018 Young et al., 2014, 2017; Paret et al., 2016; Nicholson et al., 2017). The different methods for extraction of the BOLD signal reported across studies were: using a percentage of the signal changes related to a baseline, applying a beta-values discrimination method, a GLM calculation, using event-related averaging of ROI, using a sliding window correlation analysis, and applying the Bayesian model. The majority of these studies (12 studies) reported using a percentage of signal change in relation to baseline, which was defined as the rest condition in all cases (Table 3a).

Both online and offline data preprocessing methods applied among studies were (Table 3b): (1) Slice timing correction, (2) Head motion correction, (3) Drift / artifact removal, (4) EPI distortion correction, and (5) Spatial smoothing. During online preprocessing, motion correction was applied in all studies whereas spatial smoothing was only reportedly applied online in four of the 19 studies (Paret et al., 2014, 2016; Li et al., 2016; Nicholson et al., 2017) but in all the studies during *post-hoc* analysis. EPI distortion correction was applied only once online (Marxen et al., 2016) whereas three studies reported offline EPI distortion correction (Koush et al., 2015; Paret et al., 2016) or at least distortion inspection (Lorenzetti et al., 2018).

### Amygdala Modulation—How Data Acquisition Parameters, Study Design, and Neurofeedback Protocol Features Relate With Results

#### Clinical vs. Non-clinical Population

Both studies that focused on Post-Traumatic Stress Disorder showed significant results of amygdala modulation when comparing its activation patterns for Regulate Condition vs. Control Condition, during the Transfer Run.

Regarding the sample of subjects diagnosed with Borderline Personality Disorder, no significant results were reported. Interestingly, the same protocol was administered to a sample of healthy subjects and described in a previous paper from the same authors (Paret et al., 2014), with significant results on right amygdala modulation during the Transfer Run.

Concerning Major Depressive Disorder, all three studies reported significant differences in left amygdala modulation during the regulation condition between the Last Run and the Transfer Run. Only one of the studies (Zotev et al., 2016) reported an effect of condition in the Transfer Run, and no differences between conditions were found in the Last Run for the experimental group, and no differences between conditions in the Transfer Run for the Control Group.

#### Single vs. Multiple Sessions

Considering a single Session (Table 4a), 12 in 19 studies showed significant differences in the percentage (%) of BOLD signal change in the amygdala in at least one of the following variable contrasts, defined by each author as an outcome measure: Regulate Condition vs. Control Condition, in the First Run, Last Run and Transfer Run; First Run vs. Last Run; Transfer Run vs. Last Run; and Experimental Group vs. Control Group. In the studies that applied more than one NF training Session, only two studies reported a significant main effect of Session [(Brühl et al., 2014): *F*_(3, 12)_ = 4.771, *p* = 0.021, (Young et al., 2017): *F*_(5, 517)_ = 2.37, *p* = 0.04].

#### Experimental vs. Control Group

Concerning group effects, there were five studies addressing the contrast between Experimental Group and Control Group (Table 4b). Two of these studies presented significant results of this contrast during the Last Run and four during the Transfer Run.

In the 10 studies that applied the protocol to a Control Group, only two of them reported the statistical outcome measures of the same contrasts within both the Control Group and the Experimental Group, with five studies reporting statistical outcomes from the contrast between Experimental Group and Control Group. Three studies (Zotev et al., 2011, 2016; Hellrung et al., 2018) reported significant differences between Groups but did not report data relative to the contrast between runs or conditions within the Control Group, which is a critical omission (Table 4a).

Two studies (Paret et al., 2014; Zotev et al., 2016) reported significant differences in the contrast between conditions in the Transfer Run in the Experimental Group but not in the Control Group, which supports the hypothesis of NF training related effects (Table 4a).

#### fMRI-NF Protocol Design

Ten data records reported the contrast between conditions (Regulate Condition and Control Condition) within the Experimental Group to address results on successful modulation: seven in the Transfer Run, two in the First Run and three in the Last Run. From these, seven records (36.84%) showed significant differences between conditions in the amygdala response (Table 4a).

From the seven studies that reported no significant results in amygdala modulation in the considered contrasts (36.84%), two of them were about Down-Regulation, two were about amygdala Up-Regulation, two were about both Up- and Down-regulation and one was about top-down connectivity regulation (**Figure 4**).

Studies that used a connectivity or MVPA approach to the feedback source did not report significant effects regarding amygdala modulation. These new methods do therefore remain exploratory and their real face value remains to be confirmed. In contrast, significant results on amygdala modulation were found in at least one of the reported measures in 11 (57.89%) of the studies that used % of BOLD signal change and had a predefined ROI as a feedback source.

#### Localizer Run

The two studies (Johnston et al., 2010; Brühl et al., 2014) that used localizer runs to define a target ROI in the amygdala reported significant amygdala modulation in at least one of the measures (Tables 4a,b). The other study that used a functional localizer but defined the target ROI in the left lateral PFC (Sarkheil et al., 2015) also reported significant effects in the amygdala ROI (spherical ROI centered in coordinates).

#### Stimuli

From the seven studies that presented non-significant results in amygdala modulation in the considered contrasts, four of them used aversive pictures as stimuli for emotion induction and three did not use any stimulus during the training protocol. Two of the studies with non-significant results in amygdala modulation applied stimuli pre-assessment and two applied stimuli post-assessment. From the 9 studies that used no stimuli for emotion induction, six reported significant effects in amygdala modulation (Figure 2).

#### Emotion Regulation Strategies

From the two studies that considered induced cognitive ER strategies, the one that used Cognitive Reappraisal reported no significant effects in amygdala modulation, whereas the one that used Reality Checking reported significant effects. Six in seven studies that instructed the participants to Recall Autobiographic Memories reported evidence of amygdala modulation. From the six studies that reported non-significant results on amygdala modulation, four studies instructed the participants with a free ER strategy (Figure 3).

#### Amygdala Lateralization

From the 19 included records, 8 studies found significant responses in the left amygdala and six studies found the same response pattern in the right amygdala. It is important to point out that one study (Zotev et al., 2011) showed a training effect in the left and right amygdala but a significant group effect only in the left amygdala. In another study (Young et al., 2014), there were training effects only for left amygdala and group effects only for right amygdala. One of the studies (Zotev et al., 2016) more directly addressed amygdala lateralization by revealing a positive association between the frontal EEG asymmetry in upper alpha band during NF training (Happy vs. Rest condition) and the average BOLD amygdala laterality. Furthermore, one of the studies (Paret et al., 2014) showed a main effect of hemisphere [*F*_(1,29)_ = 19.012, *p* < 0.001].

Among the 8 studies that reported LA significant responses, five were about Up-Regulation of the brain region's activity, two were about Up- and Down-Regulation and only one was about Down-Regulation. In contrast, from the six studies that reported RA significant responses, three were about Down-Regulation and three about Up-Regulation (Figure 4).

#### Data Acquisition Parameters

Studies that did not report significant results in any of the contrasts related to amygdala modulation were identified and screened to find a common study/protocol/data acquisition or analysis. We found no common feature in any of the previously mentioned domains for the seven studies. Nevertheless, it is possible to point to differences in protocol features in three of the non-significant results' studies when comparing these with studies that reported significant outcomes. Study #5 (Li et al., 2016) reported one of the highest values of slice thickness [3.5 mm—although Lorenzetti et al. (2018) used 3.75 mm and found a significant contrast]. Three in four of the studies reporting non-significant results in all the considered contrasts (Studies #4, #10, #12) also presented atypical data acquisition parameters values in relation to the successful studies (Koush et al., 2015; Sarkheil et al., 2015), namely, a TR < 2,000 ms (TR = 1,100 ms; TR = 1,000 ms and TR = 1,500 ms; respectively). Additionally, one of the studies that reported non-significant amygdala modulation outcomes (Koush et al., 2015) reported the smallest in-plane resolution (1.8 × 1.8 mm) (Figure 5).

Regarding FA, seven (Zotev et al., 2011, 2014, 2016, 2018; Young et al., 2014; Hellrung et al., 2018; Lorenzetti et al., 2018) of the nine studies that used a FA of 90° reported significant results in at least one of the contrast measures (Tables 4a,b), but only three (Posse et al., 2003a; Paret et al., 2014; Nicholson et al., 2017) in nine when FAs below 85° were used (Figure 5; Table 3a).

Of the 12 studies with significant amygdala modulation in at least one of the measures, seven studies (#1, #6 #14, #16, #17, #18, #19) used parallel imaging acceleration (particularly, SENSE). These results are quite preliminary and the relation of this parameter with neuromodulation effects in the amygdala remain unclear (Zotev et al., 2011, 2014, 2016, 2018; Brühl et al., 2014; Young et al., 2014; Lorenzetti et al., 2018) (Table 3a).

#### Online and Offline Data Processing

Importantly, none of the studies that used anatomical templates or anatomical parcellations to define the ROI for real-time NF reported significant results of amygdala modulation in any of the contrasts. Moreover, from the 12 studies that reported significant amygdala modulation, one of the studies defined the target ROI for rtfMRI-NF with a diameter length between 5 and 9 mm, while seven of the studies reported a diameter between 14 and 20 mm.

Finally, all studies applied motion correction methods during online and offline pre-processing of fMRI data. Concerning reporting on online spatial smoothing, the same number of studies (2) with or without significant result, was found. On the other hand, only one of four studies that applied offline spatial smoothing of 8 mm reported significant amygdala modulation (Table 3b).

## Discussion

We systematically reviewed and analyzed the effects of rtfMRI-NF concerning neuromodulation of the amygdala during ER training. The efficacy of rtfMRI-NF training for amygdala's modulation in the reviewed studies was evaluated by comparing % of BOLD signal change in the targeted region using different and various outcome measures across studies, in addition to evaluating behavioral performance and psychometric outcome measures, which was not the focus of our review. In general, we found evidence for amygdala modulation during rtfMRI-NF training, although the use of different outcome measures across studies to infer on the success of NF intervention precluded a quantitative meta-analysis. This was nevertheless an important conclusion to report.

In the next sections, the results will be discussed using as guideline the research questions defined in the Introduction section.

Main question: “How does the amygdala respond to emotion regulation training using neurofeedback?”

Overall, the studies point to a possible modulation of amygdala's BOLD signal during one single Session of NF-training. There is replicated evidence of selective targeted up-regulation of the left amygdala during the recall of happy autobiographic memories in depressed (Young et al., 2014, 2017; Zotev et al., 2016) and healthy individuals (Zotev et al., 2011, 2014), during cognitive reappraisal (Sarkheil et al., 2015) and reality check (Brühl et al., 2014) in healthy controls, and in PTSD (Nicholson et al., 2017; Zotev et al., 2018). The answer to this question seems therefore to be positive, at least from the best-controlled (clinical trial) studies. Despite that, there is substantial variability between studies, study characteristics and design, protocol structure and, in particular, the outcome measures to assess BOLD signal changes in amygdala activity, which precludes identifying the optimal protocol features for rtfMRI-NF applied to Emotion regulation training. These findings suggest that there are still important limitations in the design of a clear conceptual framework of NF-training research.

Question (1.1) What are the outcome measures used among studies?

The definition of outcome measures is one of the most debated topics in the field of NF. How can we measure the success of a NF training? Should we focus on clinical outcomes or brain signal changes? How do we study the explanatory power of the protocol features? Currently, there is still no consensual solution for this problem.

The analysis of magnitude of effect sizes (i.e., meta-analysis) is suitable to test the efficacy of a treatment/experimental procedure when the results extracted from multiple experiments share a similar design and test one (or equivalent) dependent variable. However, as in a recently published systematic review regarding NF training (e.g., Rogala et al., 2016), the designs and outcome measures used in the studies reviewed here did not meet these criteria. In fact, the various statistical outcome measures reported among studies were not comparable. Accordingly, the authors reported results using different statistical methods to analyze contrasts between distinct experimental variables and considered different moments of the training protocol.

Trying to overcome some of these limitations, Rogala et al. (2016) suggested a novel and interesting strategy for the success quantification: they individually qualified the training results as a failure (0) or success (1) based on the statistical measures used by each study. Training was considered successful when at least one of the multiple statistical comparisons performed in a study was significant; then, non-parametric tests were used to estimate the correlation of each training factor with the training success scores. This is an interesting approach that allows the analysis of associations between variables and results. Nevertheless, the level of reliability of this methodology is questionable. Categorizing an intervention that presents a significant effect as successful, while not considering non-significant results in other key contrasts, most likely leads to the overestimation of the success of an intervention—and this may contribute to a bias in the results. These non-specific factors (variables associated to non-significant results) are often undervalued in NF-training designs, with statistical data sometimes being omitted. This is unfortunate as it complicates the interpretation of the results.

Concerning the reviewed studies, if we isolate the 12 studies that show significant results in the reported outcome measures and which were considered for this analysis, we conclude that none of these measured all of the important dimensions: comparison between Experimental and Control Groups, comparison between Training NF Runs and comparison between Last Run and Transfer Run. In fact, four of the selected studies showed significant results in two of the reported measurements but they did not have a Control Group; five showed only significant results (both regarding Transfer Run and the comparison between groups) but did not report data contrasting different moments of the Training (NF Runs); three studies found significant results in all the reported statistical measurements, but did not perform a contrast between the Last and an additional Transfer Run.

This great variability in the statistical outputs implies that it is currently not possible to objectively meta-analyze the effectiveness of the NF technique in amygdala modulation when comparing such different statistical outcome measures. Nevertheless, an extensive description and critical appraisal of the results is presented in the next sections.

Questions (1.2) “Are there sustained brain changes in the amygdala as a result from neuromodulation by rtfMRI-NF training across runs (within Session)?”

In most studies, results of the contrast between different moments of a Single Training Session were presented. Some of them reported the differences between the Regulate Condition and the Control Condition in the Last Run whereas others presented the contrasts of the same variables considering the Transfer Run.

Crucially, the majority of studies did not present the contrast between Regulate and Control Condition, a central contrast to ensure that the effect observed in amygdala activity was due to the induced experimental manipulation, and not to a general task involvement. This led to the conclusion that, despite evidence of amygdala activity modulation given by the significant results reported by the studies, there was clear variability and contradictory findings within and between studies, as well as missing crucial information. Therefore, there is no clear evidence of modulation within the same Session.

It is important to discuss the different results that were found between different clinical and non-clinical populations. The study describing results of rtfMRI-NF training in participants with Borderline Personality Disorder showed no significant results on amygdala modulation in any of the indicated measures, although the same authors had previously found significant effects in healthy participants during the Transfer Run (Paret et al., 2014). This suggests that clinical conditions affect the efficacy of rtfMRI-NF training, raising additional questions on the neural underpinnings of the effectiveness of this technique. On the other hand, one of the studies focused on Major Depressive Disorder and showed a significant effect of condition in the Transfer Run but no effect of condition in the Last Run (Zotev et al., 2016). This is an unexpected result, since the ability to neuromodulate the amygdala during a Transfer Run is expected to reflect improvements achieved during NF training.

Question (1.3) “Are there sustained brain changes as a result of fMRI-NF neuromodulation training across different sessions?”

Only six studies (31.58%) included more than one Training Session in the research protocol. Of these, only two reported an effect of Session. Therefore, again, there were not enough data to respond to the reliability of using modulation across Sessions to prove the efficacy of NF training. Moreover, one should also consider the effects of direct NF from those of the mental training between Sessions (Subramanian et al., 2011). Non-voluntary carry-over effects within- and across-Session should not be considered just as confounds but should instead also reflect positive non-voluntary learning effects of NF training (e.g., Rieger et al., 2018).

Question (1.4) Were the brain changes only visible in the Experimental Group (compared to the Control Group)?

The inclusion of a Control Group is a critical point to discuss as it allows the distinction between observed effects due to NF training manipulation (only visible in the Experimental Group) and observed effects more likely explained by other confound non-controlled variables (in case there are no Experimental vs. Control Group differences) (Aliño et al., 2016). Accordingly, the manipulation effect should occur only in the Experimental, not in the Control Group. However, in the reviewed articles, not all of the studies that included a Control Group reported between-group comparative results, nor even the outcomes of contrasts within the Control Group. This introduces a relevant report bias.

Question (1.5) Is the amygdala response lateralized?

Overall, it was possible to detect a tendency for the Up-Regulation protocols to result predominantly in left amygdala modulation, whereas Down-Regulation protocols more effectively modulated the right amygdala. This tendency was reported in previous studies which showed more right-lateralized activity associated with the Down-Regulation of emotions (Paret et al., 2014; Morawetz et al., 2016). Accordingly, the literature points out that the right amygdala is mainly involved in the automatic processing of emotions and left amygdala in the cognitively controlled emotional processing (Gläscher and Adolphs, 2003; Dyck et al., 2011). Published evidence supports the explicit processing lateralization of the amygdala (e.g., Zotev et al., 2016).

Question (2.1) Which stimuli were used to induce emotional states and how effective were they?

In almost half of the studies, the authors chose not to use any kind of stimuli for emotion induction. In these cases, the ER strategy applied was either autobiographic memory recall or a free and subjective strategy. In those that employed emotional stimuli, visual material from international picture systems was mainly used (e.g., negative emotional faces of anger and fear used to up-regulate amygdala activation).

Statistical significance of effects was present or absent irrespective of the use of stimuli for emotional induction. Therefore, the relationship between training efficacy and presence/type of stimulus presented is unclear. Morawetz et al. (2016) suggested that the outcome of ER appears to be unaffected by stimulus material. However, the interaction of stimulus presentation with induction strategy has to be taken into account when considering the role of stimuli as supporting modulators of amygdala activity: changing the interface from visual to other modality might affect neuromodulation across subjects, but more studies on this are required. Full examination of this question would require a within subject design.

Importantly, the amygdala is known to rapidly adapt its response to stimuli, in particular emotional stimuli (Breiter et al., 1996). Habituation to stimulus effects may diminish the ER and training effects in the studies with a repeated-stimulus design (Brühl et al., 2014). Therefore, one should consider this effect in an experimental design and/or in data analysis (e.g., Blackford et al., 2012). Designing shorter blocks (Blackford et al., 2012), and ensuring that each stimulus presented in each Session is not seen previously may primarily counteract possible habituation effects (Brühl et al., 2014; Koush et al., 2015). Moreover, one should also consider the implications of traditional “boxcar” paradigms for modeling amygdala activation (Blackford et al., 2012; Sladky et al., 2012; Plichta et al., 2014), as neurofeedback approaches usually employ this type of design. Block duration can be optimized by tuning according to the temporal time course of habituation as determined experimentally, or by modeling amygdala response parameter estimates per subject for each stimulation block separately (Sladky et al., 2012; Plichta et al., 2014). Another amygdala habituation prevention strategy associated with successful NF interventions is the inclusion of a Count Condition interspersed with the ER Condition (Zotev et al., 2011, 2016; Young et al., 2014, 2017; Yuan et al., 2014). This non-emotional task is cognitively engaging, allowing for amygdala disengagement after NF up-regulation and before the following NF up-regulation block.

Question (2.2) Which Emotion Regulation strategies were the participants instructed to use and how effective were they?

From the 10 studies that instructed their participants to apply a free ER strategy, four studies reported no significant results.

Within the studies that defined a strategy *a priori*, in those that used cognitive ER strategies, distinct effects were found: studies that used Cognitive Reappraisal as ER strategy reported no significant effects in amygdala modulation, whereas when Reality Checking was used, significant effects arose. Finally, the majority of studies that used Recall of Autobiographical Memories as the ER strategy reported significant effects. There is indeed evidence of effective modulation of amygdala activity using positive autobiographical memories, both in healthy subjects as in Major Depressive Disorder (Young et al., 2018).

Despite the apparent efficacy of ER cognitive strategies like Cognitive Reappraisal and Reality Checking, only two studies instructed and trained participants to apply them, one each. Given that nearly half of the studies with no predefined ER strategy showed significant results, this points to an important issue that requires definition, as the use of implicit or explicit mental imagery strategies for self-regulation is a current topic of debate in the literature. While explicit strategies direct the individual for a specific mean of self-regulation, implicit approaches provide no information and, therefore, allow participants to explore different strategies (Sulzer et al., 2013). However, it seems that implicit approaches increase variability in the studies, hindering the inference of causal relationships. In fact, the present data corroborates this assumption: studies in which the participants were instructed with a defined ER strategy, especially when they had the chance to previously practice this strategy, point to a more reliable positive effect than studies with a subjective and free regulation strategy.

Question (2.3) How do fMRI acquisition parameters and analysis plans relate to the identification of training effects?

The collected evidence suggests a trend for successful results in the presence of the following features: (a) low slice thickness, (b) TR not < 2,000 ms, and (c) the use of denoising methods such as drift and artifact removal and head motion correction during data preprocessing (all studies did motion correction either online and offline). Results for slice thickness conform to previous studies that have reported robust bilateral amygdala activation for slice thickness of 2 mm instead of 4 mm (Bellgowan et al., 2006; Morawetz et al., 2008), and also with better SNR at 3 T for slice thickness of 2–2.5 mm (Robinson et al., 2004) or 3 mm (Merboldt et al., 2001), compared to 4–6 mm thick slices (Robinson et al., 2004). Moreover, successful modulation was obtained in most of the studies that used TR = 2 s, which has to be considered in light of previous reports of physiological confounds in BOLD changes with TRs of this magnitude (see Caballero-Gaudes and Reynolds, 2017 for a review). These results suggest that the use of shorter TRs, which at the same time imply less brain coverage (Caballero-Gaudes and Reynolds, 2017) and introduce other type of noise (Zhao et al., 2000), may not be necessary and that other parameters may be more critical.

It is important to consider that acquisition sequences and particular parameters all involve tradeoffs, which need to be considered when evaluating ultimate signal to noise ratios (SNRs) and BOLD sensitivity. This can be achieved for instance with the use of a highly sensitive MR signal detector such as an RF arrayed head coil (Fujita, 2007), the concomitant measurement of physiological signals for signal denoising, and of acceleration techniques to achieve the best tradeoff between spatial and temporal resolution and respective SNRs (for a thorough revision of fMRI parameter recommendations for amygdala measurements please see Guidelines section below).

The use of a functional localizer is an alternative to the anatomy-based approach (coordinates combined with geometrical ROIs, atlas-defined regions, manual tracing or automatic parcellation) (Sulzer et al., 2013) and has both advantages and disadvantages. Anatomical definitions are usually preferred for well-defined brain regions (e.g., amygdala, basal ganglia structures), to minimize interindividual variance. The use of a functional localizer may allow for better functional contrast guided approaches and reduced partial volume effects, due to non-significant activations that may occur within anatomically defined ROIs (Weiskopf et al., 2007). In this review, when defining the amygdala as the target for NF, few studies (only two) used a functional localizer to define the target ROI. In most cases, specific ROIs were anatomically defined based on atlases' coordinates previously reported in task-related studies or on macroscopic anatomical landmarks. One may point out that functional selection of task-specific voxels in the amygdala region may end up targeting different amygdala nuclei, each one with specific connections and functions (Roy et al., 2009; Salzman and Fusi, 2010). This individual variation in voxel selection, or the inclusion of voxels outside the amygdala, may explain inconsistent results (in our review, 50% of the studies employing localizers lacked significant results). Nevertheless, a combination of the two approaches may be helpful. For instance, some of the studies targeting the amygdala used a “best voxel selection” tool implemented in Turbo-Brain Voyager (Brain Innovation, Maastricht, Netherlands) to select task-related voxels within predefined anatomical masks (e.g., Paret et al., 2014, 2016; Nicholson et al., 2017). This tool ensured that the same number of voxels were used for signal extraction across subjects, while still restricting it to the anatomically expected location (Nicholson et al., 2017).

New techniques also arose to potentially overcome some of these limitations. Therefore, when analyzing the evidence for ER NF, it is important to differentiate traditional activation studies and studies employing new techniques such as multivariate analysis (e.g., MVPA) and connectivity analysis (e.g., effective connectivity using DCM). The later allows for strengthening of functional connections between regions, respectively (Thibault et al., 2018). However, approaches using MVPA classification results (decoding positive or negative emotions) or connection strength (in DCM) for feedback are still at their infancy. In fact, most of the studies in this systematic review focused on modifying brain activity within a targeted ROI, the amygdala. But the use of connectivity-based NF may introduce new ways to explore NF training in ER as the amygdala belongs to a network of regions contributing to ER (Thibault et al., 2018) and these techniques allow modifying the connectivity of a targeted network (Watanabe et al., 2017; Thibault et al., 2018). Unfortunately, connectivity measures are still mostly used as *post-hoc* readouts and not as NF signals. In the same manner, multivariate analysis may facilitate online classification for use in NF. Instead of relying on GLM NF, which is based on time series fluctuations and averaged amplitude of fMRI voxels in localized brain regions, it can take advantage of information from distributed voxel patterns (LaConte et al., 2007; LaConte, 2011; Sulzer et al., 2013; Watanabe et al., 2017). Data driven methods such as multivariate analyses based on machine learning can identify distributed activity patterns and may be particularly useful when individual variations in NF strategies are used (LaConte, 2011). Moreover, multivariate methods and ROI-based methods can be used in a complementary manner (LaConte, 2011; Sulzer et al., 2013). It was therefore surprising that in this systematic review employing these new methods over the traditional GLM-based approach did not present significant effects concerning successful rtNF training (Koush et al., 2015; Li et al., 2016).

Question (2.4) Is there any potential bias created by the NF protocols?

For an overall judgement of the general internal validity of the results of the reviewed literature, key methodological limitations and an analysis of the (in)consistencies of those results were addressed. Biases can lead to underestimation or overestimation of the true intervention effects. To investigate the risk of bias, the following categories were analyzed: selection bias, performance bias, attrition bias and reporting bias (Higgins and Green, 2011).

Since 14 of the 19 studies did not completely describe the participants' selection process, there is risk of selection bias. Additionally, one study did not refer the gender of the participants, one study recruited only female participants and one study recruited only male participants, which prevents generalization to the whole population, although reduces the variability due to gender issues. Additionally, not all the studies reported blinded or masked processes of participants' assignment into groups. From the 11 studies that included a Control Group, only six referred a random and blinded allocation of participants into their group. This established a risk of bias within the seven studies that may not have performed an adequate and concealed allocation process.

Regarding the completeness of data related to study information, there were no reported participant dropouts, with five studies reporting justified participants' exclusion (varying from one to four participants). The reasons for exclusion were: technical errors, large head motion, incomplete data for statistical analysis and participants falling asleep during the task. Regarding completeness of data from statistical outcome measures (in which concerns amygdala modulation), from the 10 studies that included a Control Group, three studies did not report comparative statistical results within the Control Group or the Experimental Group or results of statistical analyses contrasting Groups. Additionally, 11 studies included a Transfer Run, but only six reported the statistics of the contrasts for this moment of the protocol and only six studies reported statistical data regarding the contrast between the Last NF Run and the Transfer Run. These omissions of statistical outcomes seem to point to potential biases across the reviewed studies, which may be considered as a risk for selective reporting bias irrespective of their overall merit.

### Guidelines for Future Neurofeedback Training Protocols for Emotion Regulation

The current discussion led us to propose a few rules-of-thumb to help develop approaches, preventing nuisance effects from uncontrolled non-specific factors.

First, it seems important to ensure the specificity of the effects found during NF training. Are the significant amygdala responses reported causally related to the NF-training or, instead, can these be better explained by other variables? An accurate definition of the ROI is essential here. A combination of anatomical and functional approaches should be preferred to ensure ROI correct definition across subjects but also task-specific voxel selection.

Although difficult to select, given the matching required, the definition of a control ROI should also be considered to ensure that the effects are specific for the target ROI (Sorger et al., 2019).

Moreover, when functionally defined, the localizer run used the same stimuli of the NF runs, which may pose a problem of stimuli habituation. NF designs targeting brain regions prone to habituation, e.g., amygdala, should specifically address habituation through experimental designs and modeling parameters to minimize these effects.

Another relevant factor for the interpretation of the causality of the effects found is the inclusion of a Practice Run and a Transfer Run in the protocols. Previous reports discussing the current frame on NF studies postulate that offline mental training between Sessions could be advantageous toward accelerating learning (Sulzer et al., 2013). Pre-post data (from Practice Run and Transfer Runs) could advance the understanding of whether and to what extent amygdala NF training can change the activation of neural circuitries of ER. Moreover, it would allow reliable protocol replications in future studies.

NF training protocols should also include a Control Group, to control for confounds in learning, behavioral and placebo effects (Sulzer et al., 2013). A Control Group allows for the demonstration of behavioral change that is directly related to rtfMRI feedback training and to establish causality and specificity. In fact, experimental designs that lack a no-feedback or sham feedback Control Group cannot dissociate whether the change in the target region's activity is due to the provided feedback or instead to other variables such as mental strategies, attention capacity, motivation or another external factor. Well-controlled protocol designs are needed since only when controlling for confounding variables can one tease apart the effects of a rtfMRI-NF intervention (for a thorough discussion on controls definition, see Sorger et al., 2019). Reporting consistent and comparable outcome measures would allow for a meta-analytical assessment of the NF efficacy. First, due to the substantial variability in the number of participants in a rtfMRI-NF study, effect sizes (Cohen's *d*) should be reported in addition to *p*-values.

Second, the inclusion of linear increase in difficulty from run to run to upregulate amygdala activation via NF is recommended, as it has been shown effective to promote a linear trend in the learning process across NF runs in the Experimental Group (Zotev et al., 2014, 2016, 2018). Finally, to understand how the amygdala responds to NF training, it would be helpful to structure the statistical analysis plan by levels of outcome measures of amygdala modulation: (1) control of brain activity with neurofeedback, and (2) control of brain activity without neurofeedback (Transfer Run). Results for amygdala modulation should therefore report effect sizes of the following contrasts: (1) Regulate vs. Control Condition (effect size of amygdala activity levels averaged across all NF runs, both for Experimental and Control groups), (2) Experimental vs. Control Group (for the Regulate Condition, and in two different moments of the Training protocol: Practice Run and Transfer Run), (3) Practice Run vs. Transfer Run (within both groups). Not performing or not reporting these contrasts or at least one of these, defined *a priori* based on a specific hypothesis, may constitute a strong handicap and places the study in a position of potential bias.

In the future, we hope to be able to isolate studies using a commonly structured protocol (including Practice Run and Transfer Runs) in order to perform group level quantitative systematic review of ER NF studies. Nevertheless, such meta-analytic approach also presents a few limitations with the additional risk of isolating one protocol structure that may not be the most effective for particular applications.

Considering methodologic aspects of fMRI, the study protocol should also define sequence parameters such as pixel size (in-plane resolution), slice thickness, TE and slice orientation adjusted to the target region. The use of TR in the order of 2 s seems to be a good compromise. In general, signal loss increases with TE (Posse et al., 2003a,b), slice thickness, and voxel size (Olman et al., 2009; Caballero-Gaudes and Reynolds, 2017). Particularly for the amygdala region, previous studies have reported results of BOLD sensitivity simulations showing better results for slice thickness equal of inferior to 3 mm (Merboldt et al., 2001; Robinson et al., 2004; Bellgowan et al., 2006; Morawetz et al., 2008). However, the choice of slice thickness should balance reduction of volume coverage, signal dephasing and loss in SNR (Young et al., 1988; Posse et al., 2003b). Both coronal (Chen et al., 2003; Robinson et al., 2004) and axial or axial-oblique (Robinson et al., 2004) section orientations were found to be optimal for amygdala acquisition, although axial orientation may be preferred as it allows for a larger brain coverage per slice (Robinson et al., 2004). Moreover, in regions particularly prone to distortion such as the amygdala, fast image acquisition methods such as SENSE and GRAPPA (Blaimer et al., 2004; Bhavsar et al., 2013) can be used to reduce geometric distortion and signal dropout (Olman et al., 2009; Bhavsar et al., 2013) although some noise enhancement has been reported (Marxen et al., 2016). The combination of parallel imaging with multi-echo EPI might compensate for this loss (Bhavsar et al., 2013), increasing BOLD signal changes in subcortical brain structures such as the amygdala. Although the concomitant use of the two methods may reduce imaging artifacts and preserve BOLD sensitivity (Bhavsar et al., 2013), one should also consider that the use of multi-echo brings limitations such as loss of spatial resolution, as smaller matrix sizes are required to increase the number of TEs that can be collected (Posse et al., 2003b). Good results have been achieved with single echo times combined with parallel imaging for the amygdala region (Kirilina et al., 2016) and successfully applied in rtfMRI-NF studies (e.g., Zotev et al., 2011). To more effectively parcel out physiological noise effects, the concomitant acquisition of heart and respiratory measures during the experiment is advised (e.g., Zotev et al., 2011, 2014, 2016, 2018; Young et al., 2014; Koush et al., 2015; Marxen et al., 2016), allowing cardiorespiratory artifact correction and therefore improving *post-hoc* processing and data analysis (Caballero-Gaudes and Reynolds, 2017). Although requiring computationally intensive fMRI processing, real-time correction of physiological noise is also possible to perform (see real-time method developed by Misaki et al., 2015).

Finally, attention should be given to prevent regional geometric distortions, decreasing the variability in the coordinates for each region. Nowadays, brain imaging software packages provide tools for distortion correction, either through acquisition of additional phase and magnitude EPI scans to map the spatial distribution of field inhomogeneities (e.g., Hutton et al., 2002) or through acquisition of an additional single-volume EPI scan with reverse phase encoding polarity to estimate a voxel displacement map (e.g., Pedersen et al., 2018). Nevertheless, this needs to be considered during data acquisition.

In sum, a rtfMRI-NF protocol and parameter selection should be defined to allow for critical comparisons that can effectively measure NF training effects. More studies are needed with a multi-visit design to proof efficacy in larger populations including patients on medication, and one should carefully weigh the pros and cons of each methodological fMRI parameter considering the region being studied before designing a rtfMRI-NF.

### Limitations and Future Work

The main limitation of this study was the sample size, as we had only 19 studies fulfilling the inclusion criteria for this review. One can say that if a less keyword selective search formula was applied we would also loose data quality and specificity in the review.

Another point worth questioning was the choice of a brain structure to modulate—the amygdala—given that other brain structures are also involved in ER. In fact, several of the present studies presented results regarding other brain regions, such as the insula (Johnston et al., 2010; Paret et al., 2014; Li et al., 2016; Nicholson et al., 2017), the anterior cingulate cortex (Johnston et al., 2010; Li et al., 2016) and the prefrontal cortex (Koush et al., 2015; Sarkheil et al., 2015; Paret et al., 2016). However, the decision to center this review on the amygdala was taken given the central role of this structure in the ER network.

For future work, if one wants to use effective ER training to address pathological processes in clinical populations, it might be relevant to understand how other brain structures respond to ER training using NF, the dependence of NF on ER methodology and tasks employed, and the specificities of the populations being studied. A recent systematic review (Zilverstand et al., 2016) highlighted brain activity differences between clinical and healthy cohorts suggesting that in clinical populations different subdomains of ER may be affected, involving different brain structures. Accordingly, hyperactivation of the amygdala seems to occur in mood disorders. Instead, hypoactivation of dACC/parietal cortex occurs in anxiety related disorders. This review (Zilverstand et al., 2016) also provides some evidence that ER deficits may not only be disorder-specific but also task dependent. It is therefore important to conduct future studies comparing the efficacy of NF training in the amygdala in mood and anxiety disorders as well as in healthy cohorts. Regardless, the choice of the brain region to modulate through NF in the clinical population should be driven by clinical neuroscience approaches. In other words, the NF target region should be defined according to the characteristic cognitive impairments of the clinical condition.

## Conclusion

Several reports in the literature on ER training through rtfMRI-based NF focus on the potential relevance of the amygdala in this process. To our knowledge, this is the first systematic review on the efficacy of rtfMRI-NF for ER using modulation of BOLD signal in the amygdala as an outcome measure of interest. However, despite the identification of positive results, it turns out that this outcome measure was heterogeneously defined across studies and arises from various and different condition contrasts. Classification of the success of NF ER modulation (potential success measures) was also often based on distinct criteria. Nevertheless, some of the positive results conformed to the high standards of clinical trials.

It is vital to find common criteria and comparable quantitative outcome measures of NF training. We identified a large variability of contrasts employed across studies to measure the effects of amygdala modulation. Standardization of effect size measures would enable meta-analysis based on the critical protocol features. An unbiased understanding of modulation mechanisms is possible, thus, allowing study designs that can determine the generalization of effects.

In conclusion, this work provides qualitative evidence for amygdala modulation during neurofeedback, but also highlights the large heterogeneity across NF studies for ER training, both of which concern experimental design and choice of measures to define the efficiency of an intervention, as well as statistical analysis plans and definition of primary and secondary outcome measures. We suggest some guidelines to reduce the heterogeneity and bias of protocols so that effect sizes can be generalized to the population in future work.

## Author Contributions

ARB designed the work, performed data collection, data analysis and interpretation, and wrote the article. IA participated in data collection, data analysis and interpretation, wrote parts of the sections and critically revised the article. BCB participated in the data analysis. MC-B designed the work, contributed to data interpretation and critically revised the article for final approval of the version to be published.

### Conflict of Interest Statement

The authors declare that the research was conducted in the absence of any commercial or financial relationships that could be construed as a potential conflict of interest.
